# MEF2 transcription factors are key regulators of sprouting angiogenesis

**DOI:** 10.1101/gad.290619.116

**Published:** 2016-10-15

**Authors:** Natalia Sacilotto, Kira M. Chouliaras, Leonid L. Nikitenko, Yao Wei Lu, Martin Fritzsche, Marsha D. Wallace, Svanhild Nornes, Fernando García-Moreno, Sophie Payne, Esther Bridges, Ke Liu, Daniel Biggs, Indrika Ratnayaka, Shane P. Herbert, Zoltán Molnár, Adrian L. Harris, Benjamin Davies, Gareth L. Bond, George Bou-Gharios, John J. Schwarz, Sarah De Val

**Affiliations:** 1 Ludwig Institute for Cancer Research Ltd., Nuffield Department of Medicine, University of Oxford, Oxford OX3 7DQ, United Kingdom;; 2 Center for Cardiovascular Sciences, Albany Medical College, Albany, New York 12208, USA;; 3 Department of Physiology, Anatomy, and Genetics, University of Oxford, Oxford OX1 3QX, United Kingdom;; 4 Department of Oncology, Weatherall Institute of Molecular Medicine, University of Oxford, Oxford OX3 7LJ, United Kingdom;; 5 Institute of Aging and Chronic Disease, University of Liverpool, Liverpool L7 8TX, United Kingdom;; 6 The Wellcome Trust Centre for Human Genetics, University of Oxford, Oxford OX3 7BN, United Kingdom;; 7 Faculty of Life Sciences, University of Manchester, Manchester M13 9PT, United Kingdom

**Keywords:** sprouting angiogenesis, MEF2 transcription factors, HLX regulation, Dll4 regulation

## Abstract

Angiogenesis, the fundamental process by which new blood vessels form from existing ones, depends on precise spatial and temporal gene expression within specific compartments of the endothelium. However, the molecular links between proangiogenic signals and downstream gene expression remain unclear. During sprouting angiogenesis, the specification of endothelial cells into the tip cells that lead new blood vessel sprouts is coordinated by vascular endothelial growth factor A (VEGFA) and Delta-like ligand 4 (Dll4)/Notch signaling and requires high levels of Notch ligand DLL4. Here, we identify MEF2 transcription factors as crucial regulators of sprouting angiogenesis directly downstream from VEGFA. Through the characterization of a *Dll4* enhancer directing expression to endothelial cells at the angiogenic front, we found that MEF2 factors directly transcriptionally activate the expression of *Dll4* and many other key genes up-regulated during sprouting angiogenesis in both physiological and tumor vascularization. Unlike ETS-mediated regulation, MEF2-binding motifs are not ubiquitous to all endothelial gene enhancers and promoters but are instead overrepresented around genes associated with sprouting angiogenesis. MEF2 target gene activation is directly linked to VEGFA-induced release of repressive histone deacetylases and concurrent recruitment of the histone acetyltransferase EP300 to MEF2 target gene regulatory elements, thus establishing MEF2 factors as the transcriptional effectors of VEGFA signaling during angiogenesis.

## Abstract (precis)

In this study, Saccilotto et al. investigated the molecular connection between proangiogenic signals and downstream gene expression during angiogenesis. By characterizing a Dll4 enhancer directing expression to endothelial cells at the angiogenic front, the authors identified MEF2 transcription factors as crucial regulators of sprouting angiogenesis directly downstream from VEGFA.

Sprouting angiogenesis, in which new blood vessels form from existing ones, is driven in response to insufficient supplies of nutrients and oxygen. This response involves an exquisitely regulated pattern of gene expression directly downstream from vascular endothelial growth factor A (VEGFA) stimulation (Hellström et al. 2007; Benedito et al. 2009). As a consequence of these stimuli, a subset of endothelial cells known as tip cells forms polarized filopodia protrusions and assumes the lead positions at the tip of each blood vessel sprout (Gerhardt et al. 2003). Neighboring angiogenic endothelial cells with greater proliferative activity, known as stalk cells, contribute to elongation, stability, and lumenization of the new sprout, while other endothelial cells maintain a quiescent state, thus ensuring the stability of the existing vasculature (Gerhardt et al. 2003).

While many factors can elicit an angiogenic response, and numerous pathways influence sprouting angiogenesis, the Notch signaling pathway is crucially required to coordinate endothelial cell behavior during vessel patterning downstream from VEGFA signaling. In particular, the specification of endothelial cells into tip and stalk cells is regulated by Delta-like ligand 4 (Dll4)/Notch signaling (Hellström et al. 2007). Higher levels of the Notch ligand DLL4 in tip cells results in increased Notch signaling in the neighboring stalk cell, which in turn actively suppresses the tip cell phenotype (Hellström et al. 2007; Jakobsson et al. 2010).

The mRNA expression patterns of many vascular genes, including *Dll4*, are precisely controlled at the angiogenic front (Claxton and Fruttiger 2004; del Toro et al. 2010; Strasser et al. 2010), supporting a role for transcriptional regulation in this process. However, the transcriptional networks that elicit this specific response to VEGFA gradients are very poorly understood. While a number of transcription factors influence angiogenesis (De Val and Black 2009), gene ablation studies often result in similar vascular phenotypes, making it challenging to ascribe specific roles to different transcription factors. Some implicated transcription factors, such as members of the ETS factor family, including ERG (Randi et al. 2009; Birdsey et al. 2015), play a role in the regulation of most endothelial-expressed genes (De Val and Black 2009), including those not expressed at the angiogenic front (e.g., Tie2), suggesting that they are unable to provide the required specificity individually. Furthermore, the lack of any characterized *cis*-regulatory elements (enhancers) that directly regulate differential expression at the angiogenic front in vivo has presented a major challenge when attempting to identify direct gene targets of transcriptional and signaling angiogenic networks. Many studies have focused instead on putative regulatory sequences identified by proximity to the core promoter. For example, multiple studies have focused on the *Dll4* promoter region (Hayashi and Kume 2008; Roukens et al. 2010; Corada et al. 2013; Lizama et al. 2015), although recent transgenic mouse and zebrafish analyses have demonstrated that this region is neither sufficient nor required for *Dll4* expression in vivo (Sacilotto et al. 2013; Wythe et al. 2013). A large body of work has now clearly determined that complex patterns of gene expression require multiple interactions between promoter and enhancer elements, the latter usually located away from the core promoter (for example, Kieffer-Kwon et al. 2013), stressing the need for a greater focus on distal enhancer regions when studying transcriptional pathways. Here we describe the first enhancer capable of directing precise, differential gene activity during angiogenic sprouting. By investigating the regulation of this enhancer, we uncovered an unexpected and essential role for MEF2 transcription factors in the regulation of *Dll4* gene expression in tip cells and in the activation of gene expression during sprouting angiogenesis more generally in both physiological and pathological vascular growth.

## Results

### The *Dll4* enhancer Dll4in3 directs expression to endothelial cells during sprouting angiogenesis

**Figure 1.**
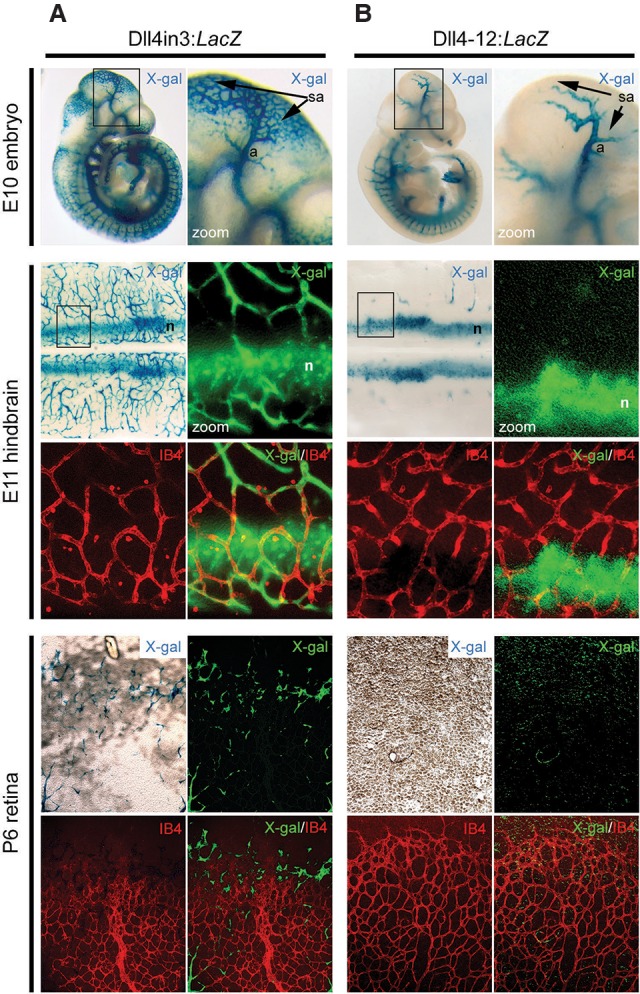
The Dll4in3 enhancer directs gene expression to endothelial cells during sprouting angiogenesis. (*A*) Representative images from Dll4in3:*LacZ* transgenic mice demonstrate enhancer activity in endothelial cells undergoing sprouting angiogenesis in the E10 embryo, E11 hindbrain, and postnatal day 6 (P6) retina. (*B*) Representative images from Dll4-12:*LacZ* transgenic mice demonstrate enhancer activity in arterial and neural tissues but no activity in endothelial cells during sprouting angiogenesis in E10 embryos, E11 hindbrains, or postnatal retinas. Enhancer activity was detected as X-gal activity (blue staining or green pseudocolor), and endothelial cells were detected by isolectin B4 (IB4) whole-mount immunostaining (red). (a) Artery; (sa) region of sprouting angiogenesis; (n) neuronal staining. See also Supplemental Figure 1.

Previously, two arterial enhancers for the Notch ligand *Dll4* have been described: one located within the third intron (referred to here as the Dll4in3 enhancer) (Sacilotto et al. 2013; Wythe et al. 2013) and the other located 12 kb upstream of the transcriptional start site (Dll4-12 enhancer) (Sacilotto et al. 2013). Similar to endogenous *Dll4*, both enhancers were active in arterial but not venous endothelial cells, an expression pattern precisely regulated by ETS, RBPJ, and SOXF transcription factors (Sacilotto et al. 2013; Wythe et al. 2013). However, endogenous *Dll4* is also expressed in tip cells leading the formation of new vessel sprouts. Consequently, we investigated whether these *Dll4* enhancers were able to direct reporter gene expression during sprouting angiogenesis. We detected Dll4in3-driven *LacZ* reporter gene activity in angiogenic vessels in a pattern closely mimicking that of endogenous *Dll4,* including expression in endothelial cells undergoing sprouting angiogenesis within the hindbrain at embryonic day 11 (E11) and in the postnatal retina (Fig. 1 A; Supplemental Fig. 1A–C). The reporter gene expression at the angiogenic front in the retina was specific and persisted throughout sprouting angiogenesis (Supplemental Fig. 1C).

Dll4in3 and Dll4-12 enhancers both contain the ETS-, RBPJ-, and SOXF-binding motifs essential for arterial expression (Sacilotto et al. 2013). Consequently, we investigated whether the Dll4-12 enhancer could also drive expression in the angiogenic sprout. However, although Dll4-12:*LacZ* activity appeared equally robust in arterial endothelial cells at both embryonic and postnatal stages, no transgene activity could be detected in endothelial cells undergoing active angiogenesis (Fig. 1B; Supplemental Fig. 1D,E). E11 hindbrains from multiple independent transgenic founders were examined to exclude the possibility of influences downstream from transgene insertion (Supplemental Fig. 1D). These results demonstrate that the Dll4in3 enhancer contains DNA sequences that convey unique transcriptional information and suggest that gene expression in sprouting angiogenesis is regulated by factors other than, or in addition to, ETS, RBPJ, and SOXF.

### MEF2 transcription factors regulate *Dll4* enhancer expression during sprouting angiogenesis in both physiological and pathological vessel growth

**Figure 2.**
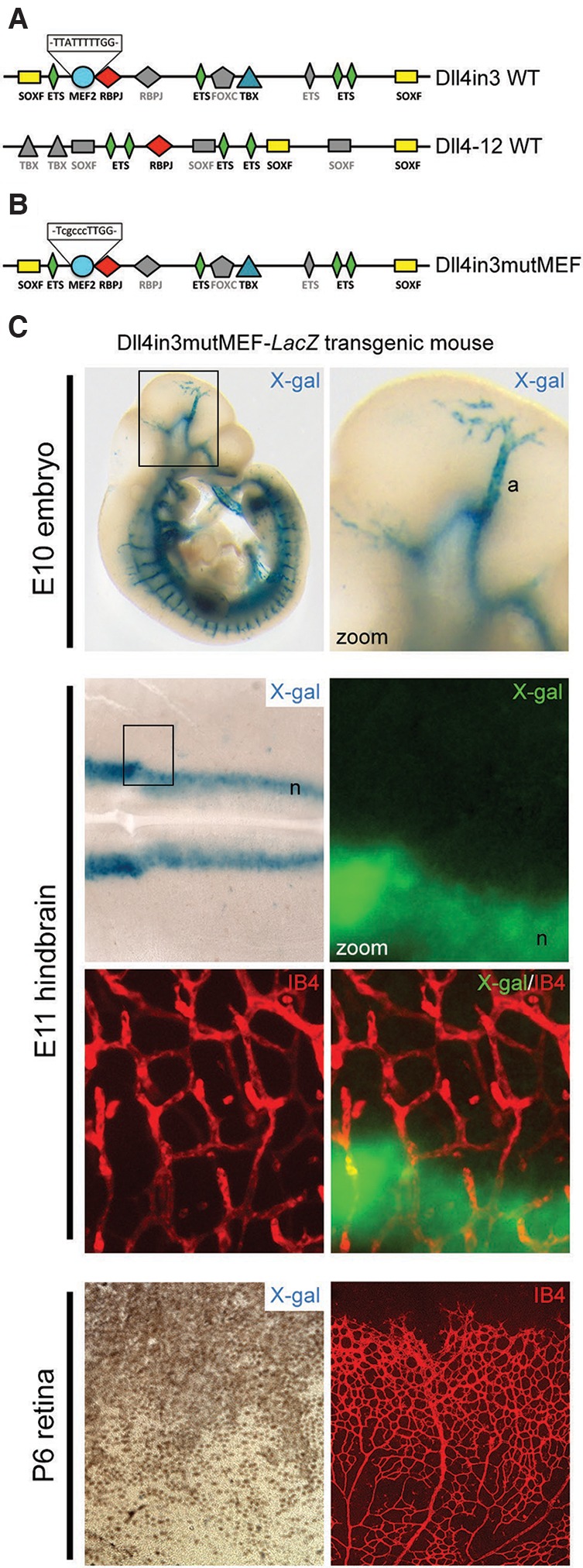
The Dll4in3 enhancer contains a MEF2-binding motif that is required for expression during sprouting angiogenesis. (*A*) Schematic representation of the binding motifs found within the mouse Dll4in3 and Dll4-12 enhancer sequences. Colored shapes represent sequences directly verified by EMSA analysis (Supplemental Fig. 2; Sacilotto et al. 2013), and gray shapes indicate consensus or near-consensus sequences that did not bind the cognate transcription factors in EMSA. (*B*) Schematic representation of the Dll4in3mutMEF enhancer sequence in which the MEF2-binding motif within the Dll4in3 enhancer has been mutated. (*C*) Representative images from Dll4in3mutMEF transgenic mice demonstrate that this enhancer does not direct endothelial expression during sprouting angiogenesis in E10 embryos, E11 hindbrains, or P6 retinas, although robust expression is detected in the arterial (a) and neuronal (n) compartments. See also Supplemental Figure 2.

A comparison of the Dll4in3 and Dll4-12 enhancer sequences demonstrated that the angiogenic Dll4in3 enhancer contained consensus MEF2- and Forkhead C (FOXC)-binding motifs that were not present in the Dll4-12 sequence (Fig. 2A; Supplemental Fig. 2A,B). The Dll4in3 MEF2-binding motif bound MEF2A, MEF2C, and MEF2D proteins in electophoretic mobility shift assay (EMSA) and chromatin immunoprecipitation (ChIP) analysis (Supplemental Fig. 2C–E; Sacilotto et al. 2013; Wythe et al. 2013), whereas the putative FOX motif did not bind FOXC proteins in EMSA and had no detectable effect on Dll4in3:*LacZ* expression at E11 (Sacilotto et al. 2013). We therefore examined the role of the Dll4in3 MEF2-binding motif in enhancer activity in transgenic mice. Although mutation of the MEF2-binding motif present in Dll4in3 (Fig. 2B) had no effect on reporter gene expression in arterial endothelial cells (Fig. 2C; Sacilotto et al. 2013; Wythe et al. 2013), loss of MEF2 binding led to an almost total loss of reporter gene expression in embryonic hindbrains and postnatal retinas during sprouting angiogenesis (Fig. 2C; Supplemental Fig. 2F,G). These results therefore indicate that the MEF2-binding motif within the Dll4in3 enhancer is essential for directing gene expression in endothelial cells undergoing active sprouting angiogenesis.

**Figure 3.**
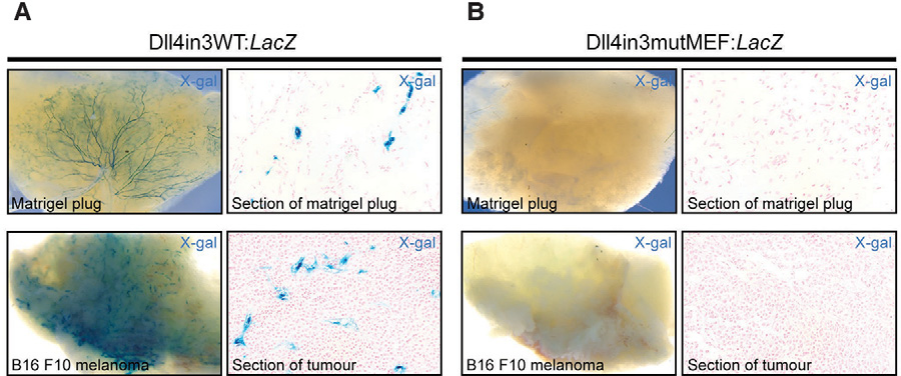
The Dll4in3 enhancer requires MEF2 binding to direct expression during adult neovascularization. (*A*) Representative images of Dll4in3:*LacZ* transgene expression during neovascularization into Matrigel plugs and B16F10 melanoma tumors demonstrate robust vascular X-gal staining. (*B*) Representative images of the mutant Dll4in3mutMEF:*LacZ* transgene demonstrate that the mutated enhancer was unable to direct any detectable reporter gene expression during adult neovascularization. See also Supplemental Figure 3.

Sprouting angiogenesis also occurs in the adult in response to physiological cues that support cycles of remodeling and repair and in pathological conditions, including cancer, where establishment of a vascular system is essential for tumour growth and metastasis (Kerbel 2008). Therefore, we examined the expression of the Dll4in3 enhancer during neovascular growth in adult mice. Similar to developmental angiogenesis, the Dll4in3:*LacZ* enhancer was able to direct reporter gene expression to new vessels during Matrigel plug and B16F10 melanoma-stimulated neovascularization (Fig. 3A; Supplemental Fig. 3A,B). As with the postnatal day 6 (P6) retina, expression was not detected in every endothelial cell (Supplemental Fig. 3A); instead, the pattern closely resembled that reported in Unc5b^*LacZ*/+^ mice, in which *LacZ* expression correlated with sprouting angiogenesis (Larrivee et al. 2007). Strikingly, mutation of the MEF2-binding motif within the Dll4in3 enhancer resulted in a total loss of reporter gene expression during neoangiogenesis (Fig. 3B; Supplemental Fig. 3C,D) despite the maintenance of transgene expression in the vasculature of some adult organs (Supplemental Fig. 3E). These results clearly demonstrate the importance of the MEF2-binding motif for Dll4 enhancer expression during both physiological and pathological angiogenesis.

### MEF2 factors regulate endogenous DLL4 expression

**Figure 4.**
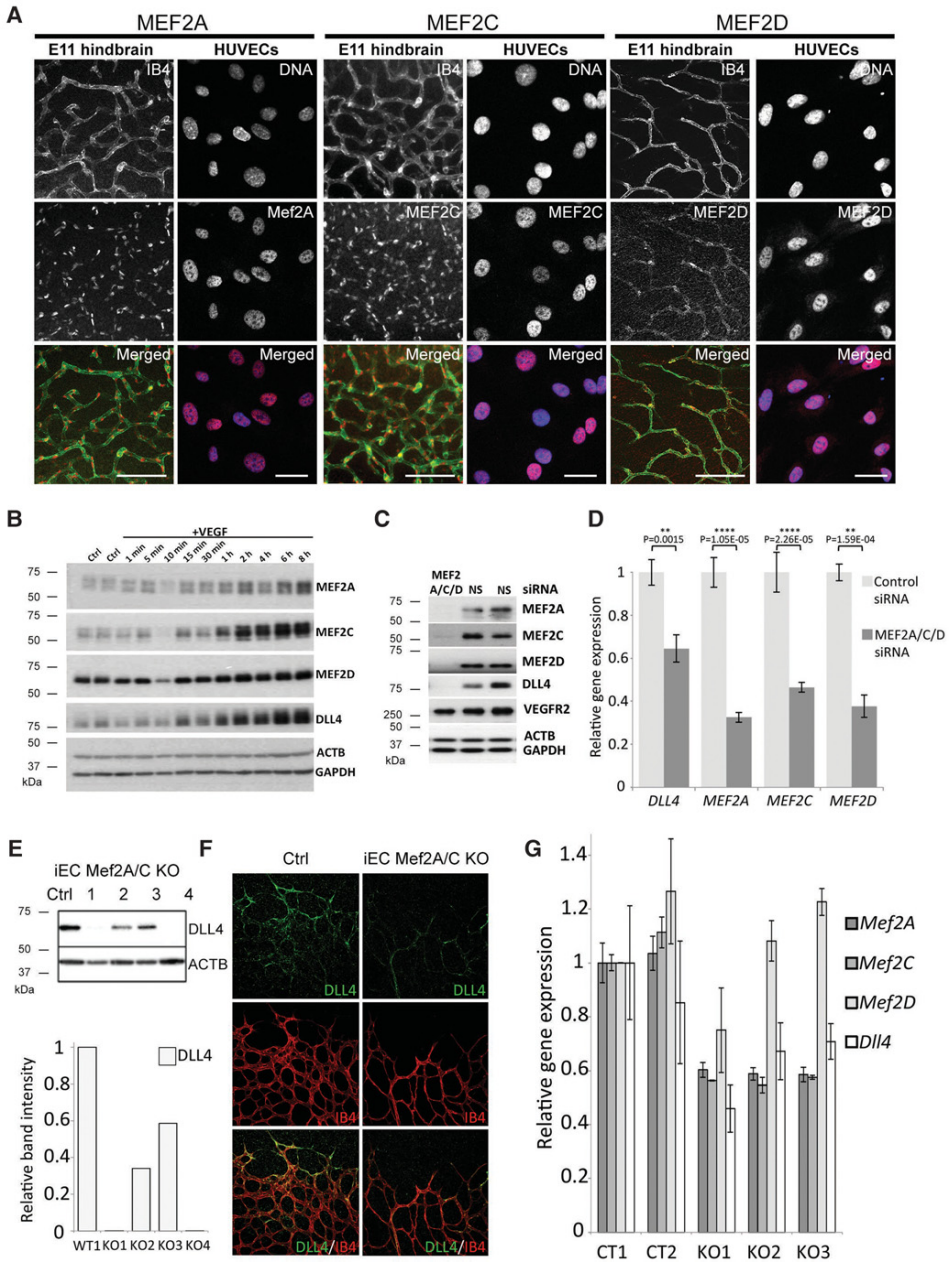
MEF2 factors regulate endogenous *Dll4* expression and tip cell identity. (*A*) Whole-mount immunostaining demonstrates expression of MEF2A, MEF2C, and MEF2D transcription factors in endothelial cells from E11 hindbrains and in HUVECs. Bars, 100 μm. (*B*) Western blot analysis of MEF2A, MEF2C, MEF2D, and DLL4 expression in HUVECs 1 min to 8 h after VEGFA stimulation. Representative of four repeats. (*C*) RNAi-mediated knockdown of MEF2A, MEF2C, and MEF2D factors by pooled siRNAs in HUVECs results in loss of DLL4 expression, while VEGFR2 expression remains unaffected. (NS) Nontargeting control siRNA. Representative of two repeats. (*D*) Relative gene expression levels of *DLL4*, *MEF2A*, *MEF2C*, and *MEF2D* analyzed by quantitative RT–PCR (qRT–PCR) after RNAi-mediated knockdown and 1 h of VEGFA stimulation. Statistical analysis was performed on four biological replicates. Error bars indicate standard deviation. (*E*) Induced endothelial cell-specific (iEC) *Mef2A/C* double knockouts (*Mef2A/C* KO) show a significant reduction in DLL4 expression, as detected by Western blot of E12 hindbrain extracts. Cre recombination was induced by tamoxifen injection in pregnant mice at E9. (Ctrl) Cre-negative; (1–4) four different iEC *Mef2A/C* knockout littermates. Note the variability in DLL4 reduction among littermates, as expected from Cre recombination in response to tamoxifen. Comparative band intensity was normalized to ACTB levels and made relative to wild type. (*F*) Endogenous DLL4 expression (green) in representative control (Cre-negative) and iEC *Mef2A/C* knockout P5 retinas at the angiogenic front. All endothelial cells were detected by IB4 whole-mount immunostaining (red). (*G*) Relative gene expression levels of *DLL4*, *MEF2A*, *MEFC*, and *MEF2D* analyzed by qRT–PCR in five different P5 lungs taken from control (Cre-negative; CT) and knockout (iEC *Mef2A/C* KO*)* pups. Three technical replicates were used for each pup. Error bars indicate standard deviation.

We demonstrated previously that RBPJ and SOXF factors, both of which are essential for Dll4in3 enhancer activity in arterial endothelium, were also required for expression of the endogenous *Dll4* gene and the acquisition of arterial fate more generally (Sacilotto et al. 2013). We therefore investigated whether MEF2 transcription factors regulate endogenous *Dll4* during sprouting angiogenesis. Although MEF2 transcription factors have been implicated in vascular integrity and endothelial cell survival (Hayashi et al. 2004; Olson 2004; Chang et al. 2006), endothelial-specific ablation of MEF2C resulted in no detected embryonic defects (Xu et al. 2012) and did not alter Dll4in3 or endogenous *Dll4* expression in early developing arteries (Wythe et al. 2013). However, Mef2C^fl/null^;Tie2^Cre/+^ mice did exhibit increased vascular sprouting after oxygen-induced retinopathy, resembling *Dll4*^*LacZ*^^/+^ mice after similar insult (Lobov et al. 2007; Xu et al. 2012). All MEF2 factors bind a similar DNA motif and can be functionally redundant (Potthoff and Olson 2007; Liu et al. 2014). Since MEF2A, MEF2C, and MEF2D were each able to directly bind the Dll4in3 MEF2 motif (Supplemental Fig. 2D,E), we hypothesized that multiple MEF2 factors may regulate *Dll4* expression. Supporting this, we detected expression of MEF2A, MEF2C, and MEF2D in endothelial cells from multiple tissues and with expression dynamics correlating to that of *Dll4* after VEGFA (Fig. 4A,B; Supplemental Fig. 4A–D). Combined siRNA-mediated knockdown of MEF2A,C and MEF2D in human umbilical vein endothelial cells (HUVECs) resulted in significantly reduced Dll4 expression after VEGFA stimulation (Fig. 4C,D; Supplemental Fig. 4E,F). Furthermore, these MEF2 knockdown HUVEC cells demonstrated a reduction of tip cell competitiveness compared with control cells in VEGFA-induced mosaic spheroid sprouting assays (Supplemental Fig. 5A,B). The same phenotype was observed in VEGFA-induced sprouting of embryoid bodies after CRISPR/Cas9-mediated ablation of Mef2A and Mef2C in embryonic stem (ES) cells (Supplemental Fig. 5C–E). This reduced competitiveness was similar to previous reports demonstrating that *Dll4*^*LacZ*^^/+^ heterozygous ES cells with reduced DLL4 levels cannot compete with wild-type cells for the tip cell position during sprouting angiogenesis (Jakobsson et al. 2010).

To investigate whether depletion of MEF2 factors had a repressive effect on *Dll4* expression in vivo, we next investigated the expression levels of DLL4 in mice after endothelial-specific induced deletion of *Mef2A* and *Mef2C* [Cdh5(PAC)Cre-ER^T2^;*Mef2A*^*fl*^^/fl^;*Mef2C*^*fl*^^/fl^, denoted as induced endothelial cell-specific (iEC) *Mef2A/C* knockout]. Although *Mef2D* was not targeted in these mice, DLL4 levels were reduced in the highly angiogenic E11 hindbrains 3 d after induction of gene deletion (Fig. 4E), and substantially less DLL4 was detected at the angiogenic front in P5 retinas 4 d after the induction of gene deletion (Fig. 4F). Analysis of whole-lung extracts confirmed the significant decrease in *Dll4* transcript levels concurrent with reduction in *Mef2A* and *Mef2C* despite the inclusion of nonvascular cells in this analysis (Fig. 4G). These results therefore support a key role for MEF2 factors in the regulation of endogenous *Dll4* during sprouting angiogenesis and consequently on the differentiation of tip cells during this process.

### MEF2 factors directly regulate many genes during sprouting angiogenesis

**Figure 5.**
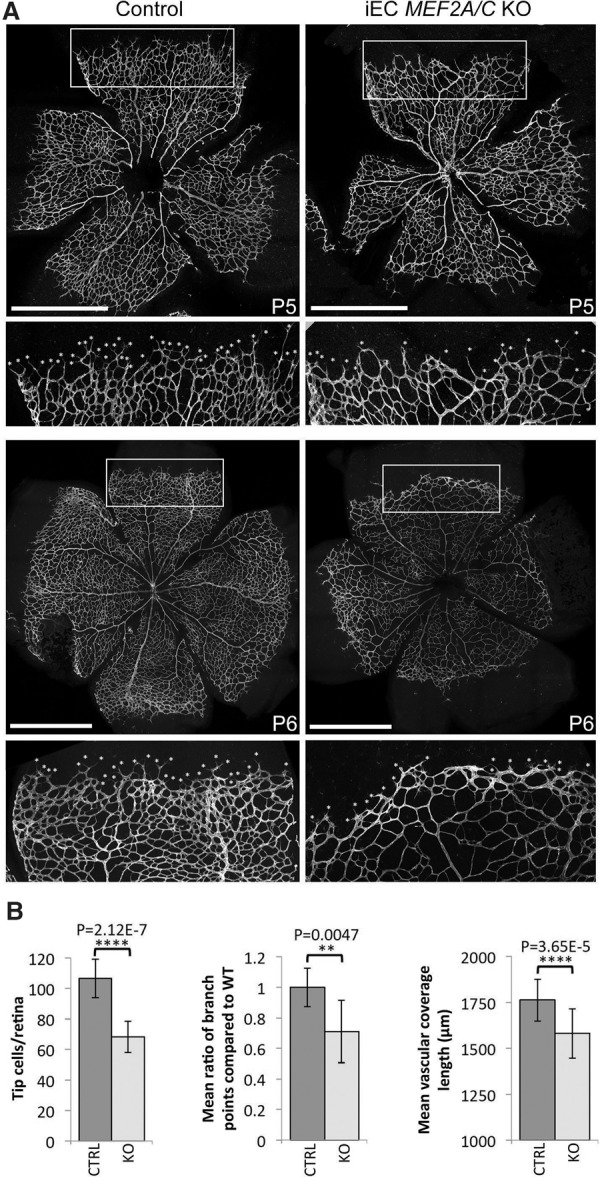
Induced endothelial deletion of *Mef2A and Mef2C* results in reduced retinal angiogenesis. (*A*) Representative P5 and P6 retinas taken from control (Cre-negative) and iEC *Mef2A/C* knockout (KO) pups and stained for IB4. Tip cells were detected by filopodia and are indicated by asterisks. The white box indicates region taken for zoom image. Bars, 1 mm. (*B*) Graphs demonstrating the mean number of tip cells, the ratio of the mean number of branch points, and the mean outgrowth length as measured by actual distance covered by the growing vessel from the center of the retina. Statistical analysis was performed on seven wild-type and nine knockout pups pooled from two different litters.

**Figure 6.**
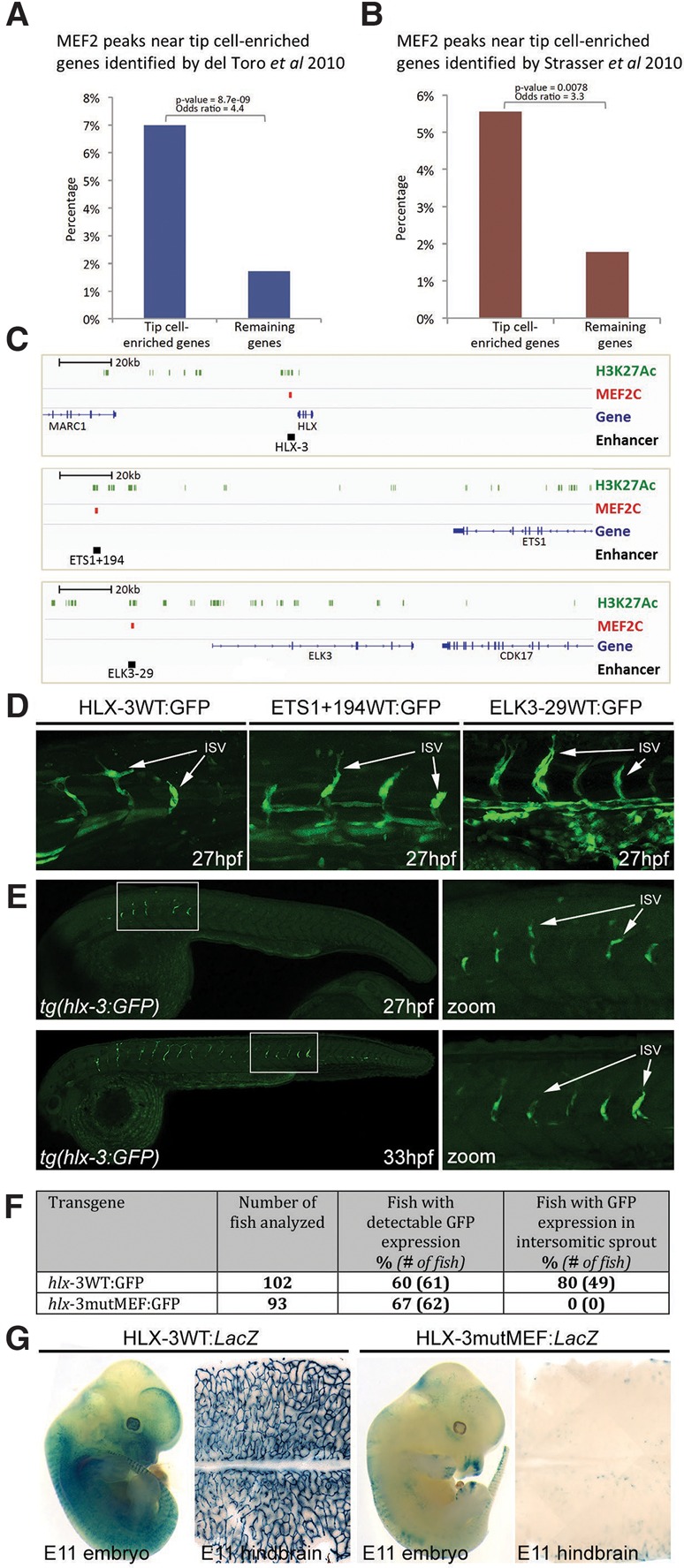
MEF2 binding is a shared feature of many angiogenesis enhancers. (*A*,*B*) MEF2C-binding peaks are enriched around 200 kb of genes associated with sprouting angiogenesis, as assessed by increased expression in the hypersprouting retina of Dll4^+/−^ mice (*P* = 8.682 × 10^−09^) (del Toro et al. 2010) (*A*) or identified through laser capture microdissection of retinal tip cells (*P* = 0.007776) (Strasser et al. 2010) (*B*). (*C*) Genomic snapshots denoting MEF2C-binding sites within the loci for HLX, ETS1, and ELK3 transcription factors. H3K27ac peaks are indicated in green, MEF2C-binding peaks are indicated in red, and novel angiogenic enhancers are indicated in black. (*D*) *tol2*-mediated mosaic zebrafish transgenic for the human MEF2C-binding peak enhancers HLX-3, ETS1+194, and ELK3-29 demonstrate enhancer-driven GFP expression in sprouting endothelial cells (green). (ISV) Intersegmental vessels. (*E*) The stable transgenic zebrafish line *tg(hlx-3:GFP)* directs expression specifically to sprouting endothelial cells in the intersegmental vessels*.* (*F*) Summary of reporter gene expression detected in 32-hpf *tol2*-mediated mosaic transient transgenic zebrafish embryos. (*G*) Whole-mount embryos and hindbrains of representative E11 X-gal-stained transient transgenic embryos expressing HLX-3 wild-type:*LacZ* and HLX-3mutMEF:*LacZ* transgenes. See also Supplemental Figures 6–8.

Although *Dll4* expression was clearly reduced after induced endothelial deletion of *Mef2A/C*, it was notable that iEC *Mef2A/C* knockout P5 retinas did not exhibit the increased levels of sprouting angiogenesis and vessel branching reported as a consequence of Notch inhibition or Dll4 reduction alone (Hellström et al. 2007). Instead, iEC *Mef2A/C* knockout retinas displayed a vascular plexus with a significant reduction of vascular density and coverage accompanied by a reduced number of tip cells at the angiogenic front (Fig. 5). These results indicate that the loss of MEF2A/C results in an overall reduction of angiogenic sprouting and point to a potential role for MEF2 in regulating vascular growth more generally. To establish whether MEF2 factors directly regulate other angiogenesis-related genes and consequently the angiogenic process more generally, we analyzed genome-wide MEF2C-binding peaks associated with enhancer-specific histone marks in HUVECs from publicly available ChIP-seq (ChIP combined with high-throughput sequencing) data (Maejima et al. 2014). Supporting our earlier analysis, the Dll4in3 enhancer region contained a robust MEF2-binding peak, whereas no similar peak was found around the Dll4-12 nonangiogenic enhancer or elsewhere in the *Dll4* locus (Supplemental Fig. 6A). Pan-genomic analysis of MEF2C-binding locations revealed a significant enrichment of enhancer-associated MEF2C binding near genes known to be up-regulated during sprouting angiogenesis (Fig. 6A,B; Supplemental Fig. 6B; Supplemental Tables 1, 2; del Toro et al. 2010; Strasser et al. 2010), suggesting a role for MEF2 factors in angiogenic gene activation beyond *Dll4* and providing a potential explanation for the decreased vascular growth seen in the iEC *Mef2A/C* knockout retinas. No enhancer-associated MEF2-binding peaks were found within 200 kb of any other Notch pathway gene, including *JAG1*, *JAG2*, *HES1*, *HEY1*, and *HEY2* (Supplemental Fig. 6C). Loci containing significant MEF2-binding peaks included the transcription factors *ETS1*, *ELK3*, and *HLX*, all of which are also implicated in the regulation of angiogenic behavior (Fig. 6C; De Val and Black 2009; Herbert et al. 2012). The human DNA sequences around these sites were tested in mosaic transgenic zebrafish at 26–30 h post-fertilization (hpf), confirming that they were bona fide angiogenic enhancers (Fig. 6D).

The MEF2C-bound enhancer element 3 kb upstream of the *HLX* gene (HLX-3 enhancer) (Supplemental Fig. 7A) was particularly notable, as *HLX/hlx* expression is unusually specific to sprouting angiogenesis (Herbert et al. 2012; Prahst et al. 2014). Furthermore, HLX expression is also known to be downstream from VEGFA stimulation through EP300 binding at the same location where MEF2C was bound (Supplemental Fig. 7A; Zhang et al. 2013). We confirmed the sprout-specific expression pattern of this enhancer in a stable zebrafish transgenic line, *tg(hlx-3:GFP)* (Fig. 5E). This enhancer contained three MEF2 sites (Supplemental Fig. 7B), the strongest of which was able to bind MEF2A, MEF2C, and MEF2D in EMSA analysis at levels comparable with the Dll4in3 MEF2 motif (Supplemental Fig. 8). Mutation of these MEF2 sites resulted in total ablation of transgene expression in both transgenic zebrafish and mice (Fig. 6F,G; Supplemental Fig. 7C,D). Together, these results suggest that MEF2 factors play a crucial role in the transcriptional activation of multiple genes associated with sprouting angiogenesis.

### MEF2 regulates tip cell genes downstream from VEGFA-mediated histone deacetylase (HDAC) derepression

Our data support a role for MEF2 factors in the activation of sprouting angiogenesis downstream from VEGFA signaling in endothelial cells. We therefore next investigated the mechanisms by which MEF2 factors are able to specifically activate gene transcription in sprouting, but not quiescent, endothelial cells. Although the transcriptional regulators of *Mef2a* and *Mef2d* in endothelial cells remain unknown, *Mef2c* transcription is known to be directly activated by ETS factors, including ETS1 (De Val et al. 2004). Therefore, MEF2 factors may lie at the top of a feed-forward loop during sprouting angiogenesis, activating the expression of *Ets* factors, which would then reinforce *Mef2* expression and also collaborate with MEF2 factors in the activation of downstream sprouting angiogenic genes, including *Dll4*. This model is supported by our observations that mRNA and protein levels of MEF2A and MEF2C notably increase after VEGFA stimulation (Fig. 4B; Supplemental Fig. 4C).

**Figure 7.**
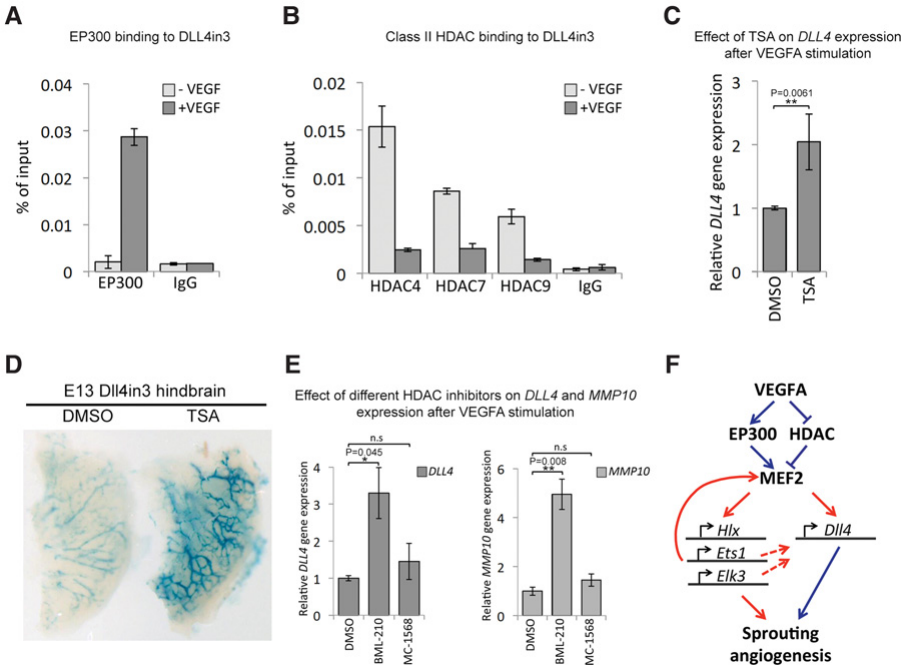
VEGFA signaling leads to activation of MEF2 transcriptional activity. (*A*) Increased EP300 binding at the DLL4in3 enhancer after VEGFA stimulation in HUVECs analyzed by ChIP. Graph is representative of four biological replicates. (*B*) Decreased class II HDAC binding at the DLL4in3 enhancer after VEGFA stimulation in HUVECs analyzed by ChIP. The graph is representative of two biological replicates. (*C*) Relative *DLL4* gene expression after VEGFA stimulation with and without trichostatin A (TSA), analyzed by qRT–PCR. Statistical analysis on four biological replicates. Error bars indicate standard deviation. (*D*) Representative Dll4in3:*LacZ* E13 hindbrains removed 24 h after in utero intracerebral injection of 10 μM HDAC inhibitor TSA or DMSO control. X-gal staining after DMSO control injection is weak, whereas more robust transgene expression was detected in hindbrains injected with TSA. (*E*) Relative gene expression levels of *DLL4* and MMP10 after VEGFA stimulation with and without treatment with small molecule class II HDAC inhibitors BML-210 and MC-1568, analyzed by qRT–PCR. Statistical analysis was performed on three biological replicates. Error bars indicate standard deviation. (*F*) Proposed model of VEGFA-mediated activation of sprouting angiogenesis via the MEF2 transcription factor family. See also Supplemental Figure 9.

However, this feed-forward loop is unlikely to be the only mechanism: The *Mef2c* enhancer itself is not specific to sprouting angiogenesis (De Val et al. 2004), ETS factors are essential for all endothelial gene expression (De Val and Black 2009; Randi et al. 2009), and MEF2 proteins can be detected in most endothelial cells (Fig. 4A; Supplemental Fig. 4A). Furthermore, ChIP analysis demonstrates that MEF2 factor binding to the DLL4in3 and HLX-3 enhancers occurs independently of VEGFA stimulation (Supplemental Fig. 9A,B), although both HLX expression (Testori et al. 2011) and DLL4 expression (Fig. 4B) are significantly up-regulated only after VEGFA stimulation. Therefore, we hypothesized that the ability of MEF2 factors to associate with transcriptional coactivators may be modified by VEGFA stimulation. The histone acetyltransferase EP300 has been shown to play a key role in transcriptional activation downstream from VEGFA in endothelial cells, where it is tightly associated with the enhancer-associated histone modification H3K27ac (Zhang et al. 2013). We therefore tested the dynamics of EP300 recruitment to the Dll4in3 enhancer and found a significant increase in binding after VEGFA stimulation (Fig. 7A). This result suggests that the ability of MEF2 factors to activate gene expression was affected directly by VEGFA-induced recruitment of EP300.

The activity of MEF2 factors in other cell types is known to be modulated by the mutually exclusive recruitment of EP300 and class II HDACs (Lu et al. 2000; Youn et al. 2000). Class II HDACs inhibit MEF2 factors by directly binding to them and consequently preventing the ability of MEF2 to recruit cofactors required for gene activation (Lu et al. 2000; Chan et al. 2003). Since VEGFA stimulation is known to directly induce class IIa HDAC phosphorylation and cytoplasmic accumulation (Ha et al. 2008a,b), we hypothesized that this may be the mechanism through which VEGFA signaling at the angiogenic front directly activates MEF2. ChIP analysis confirmed a significant decrease in binding of the class IIa HDACs HDAC4, HDAC7, and HDAC9 to the DLL4in3 enhancer after VEGFA stimulation (Fig. 7B). Furthermore, general inhibition of HDAC activity by trichostatin A (TSA) resulted in a significant increase in *DLL4* transcription in HUVECs (Fig. 7C) and Dll4in3 enhancer activity both in vivo (Fig. 7D) and ex vivo (Supplemental Fig. 9C). To confirm that the repressive activity of HDACs on *DLL4* expression occurred directly via MEF2 interaction, we tested the effects of two different small molecule inhibitors of class IIa HDACs. BML-210 works by specifically targeting and disrupting the MEF2:HDAC complex (Jayathilaka et al. 2012), whereas MC-1568 instead selectively stabilizes this complex (Nebbioso et al. 2009). Supporting the hypothesis that MEF2:HDAC binding directly inhibits *DLL4* expression in nonangiogenic cells, treatment with BML-210 resulted in a significant increase in *DLL4* transcription that is mirrored by that of *MMP10*, a known MEF2 target regulated by MEF2:HDAC interaction (Fig. 7E; Chang et al. 2006; Ha et al. 2008a). Conversely, treatment with MC-1568 did not increase *DLL4* and *MMP10* expression (Fig. 7E). Other genes known to be up-regulated or down-regulated by TSA in the presence of VEGF (Rafehi et al. 2014) but without a MEF2C-binding peak within 200 kb were not significantly affected by these compounds (Supplemental Fig. 9D).

In conclusion, these results support a model (Fig. 7F) in which VEGFA-triggered release of repressive HDACs and concurrent recruitment of the activating histone acetyltransferase EP300 to MEF2-bound enhancers results in MEF2-activated transcription of immediate downstream angiogenic target genes, including multiple ETS factors. This further reinforces the expression of *Mef2* genes though a feed-forward loop, enhances the angiogenic transcriptional cascade, and drives the high levels of *Dll4* expression required to form a leading tip endothelial cell. This activation of Notch signaling then triggers the lateral inhibition and guided vascular patterning essential for sprouting angiogenesis.

## Discussion

It is well established that endothelial sprouting is controlled by VEGF signaling (Gerhardt et al. 2003). However, VEGF receptors use multiple signal transduction pathways and transcriptional effectors to obtain strikingly different vascular outcomes, and therefore the manner in which high levels of VEGFA establish the precise gene expression patterns required for sprouting angiogenesis has been a key question in vascular biology. In this study, we demonstrate that VEGFA-induced gene expression in endothelial cells at the angiogenic front is downstream from MEF2 factor-driven transcriptional activation. In addition to their roles in the endothelium, MEF2 factors are widely expressed in nonvascular tissues and play crucial roles in the development of cardiac and skeletal muscle, bone, neural crest, and T cells (Potthoff and Olson 2007). Consequently, it is likely that they collaborate with other transcription factors to achieve endothelial-specific responses. One potential partner is the ETS family of transcription factors. All angiogenic enhancers characterized in this study contained multiple ETS motifs in addition to MEF2 sites. Furthermore, loss of ETS motifs ablated Dll4in3 expression in both arteries and sprouting angiogenesis (Sacilotto et al. 2013; Wythe et al. 2013), suggesting that ETS factors cooperate with MEF2 factors in the vasculature. This is supported by the observation that ETS factors are both transcriptional activators of and activated by MEF2C (Fig. 6; De Val et al. 2004). Of note, the highly endothelial-expressed ETS transcription factor ERG has been implicated in the regulation of *Dll4* in arteries downstream from VEGFA (Wythe et al. 2013) and in vascular growth, including in the angiogenic retina (Birdsey et al. 2015). However, it is unlikely that ERG or other ETS factors regulate angiogenic gene expression alone: Unlike MEF2 motifs, ETS-binding motifs are found in all known endothelial enhancers and promoters regardless of expression pattern within the vasculature (De Val and Black 2009), and ERG itself is expressed widely throughout the endothelium (Randi et al. 2009). Furthermore, we demonstrate here that Dll4in3 and HLX-3 enhancers with intact ETS motifs were unable to drive angiogenic expression after MEF2 motif ablation, although Dll4in3 was still able to drive arterial expression. Similarly, the wild-type Dll4-12 enhancer containing multiple functional sites for ETS but no MEF2 sites was unable to drive anything other than arterial expression. These results therefore instead support a model in which MEF2 factors cooperate with ETS factors, with ETS providing essential endothelial expression information, and MEF2 contributing to angiogenic sprout specificity.

The SOXF family of transcription factors may also play a role in MEF2-driven angiogenic gene activity. Unlike the widely expressed ETS and MEF2 factors, the SOXF factors (SOX7, SOX17, and SOX18) are primarily restricted to arterial and angiogenic endothelial cells in mammals (Corada et al. 2013; Lee et al. 2014). SOXF factors have already been implicated in the regulation of Dll4/Notch signaling (Corada et al. 2013; Sacilotto et al. 2013), but their precise role at the angiogenic front is not yet established. SOXF binding in combination with ETS factors is likely not sufficient for angiogenic expression, as multiple enhancers, including Dll4-12 and Ece1, robustly bind SOXF and ETS factors but do not drive expression during sprouting angiogenesis (Robinson et al. 2014). However, loss of SOX17 affected sprouting angiogenesis in the retina (Corada et al. 2013; Lee et al. 2014), and a role for SOXF factors in combination with MEF2 cannot be ruled out: The Dll4in3 MEF2 motif is adjacent to a functional SOXF-binding motif, and MEF2C is known to directly interact with SOX18 (Hosking et al. 2001). It would therefore be fascinating to directly compare the global binding patterns of SOX7, SOX17, and SOX18 with MEF2 factors in endothelial cells and establish the role of SOXF-binding motifs in our newly identified set of MEF2-dependent angiogenic enhancers.

In addition to collaborative binding with other transcription factors, our data also indicate that the specificity of MEF2 activation to sprouting angiogenesis is modulated by the removal of repressive class IIa HDAC binding and recruitment of EP300 downstream from VEGFA. However, MEF2 factors are able to confer signal responsiveness downstream from multiple signaling pathways. For example, multiple calcium-regulated protein kinases are able to modulate HDAC binding to MEF2 factors, and phosphorylation by MAP kinases can activate MEF2 (Potthoff and Olson 2007; Ge et al. 2009). The latter is particularly notable given that recent work implicates ERK as a specific effector of Vegfa signaling in the induction of angiogenic genes during sprouting (Shin et al. 2016). Furthermore, research in skeletal muscle also demonstrated that the MEF2 proteins can compete with NICD for binding to the MAML coactivator (Shen et al. 2006), suggesting that high levels of NICD in stalk cells may also have a repressive effect on MEF2 factors in the endothelium, reinforcing the lateral inhibition. Consequently, it is very likely that MEF2 transcriptional activity is modulated by a complex combination of transcriptional, translational, and post-translational modifications, all of which contribute to the gene expression pattern downstream from VEGF receptor signaling.

Previous work has already implicated MEF2 transcription factors in the maintenance of vascular integrity via HDAC7-mediated MMP10 repression and downstream from BMK1 (Hayashi et al. 2004; Olson 2004; Chang et al. 2006). Although these reports principally focused on MEF2C at least in part due to the cardiovascular phenotypes reported in the global MEF2C-null mice (Lin et al. 1998; Bi et al. 1999), endothelial-specific ablation of MEF2C resulted in no clear embryonic vascular defects (Xu et al. 2012). This result indicates that the vascular defects seen in the global MEF2C-null mice were principally downstream from cardiac dysfunction and suggests that MEF2 factor redundancy in the vasculature extends beyond the regulation of *Dll4*. This may mirror the situation in skeletal muscle regeneration, where an absolute requirement for MEF2 was revealed only after compound deletion of *Mef2A*, *Mef2C*, and *Mef2D* (Liu et al. 2014). However, although loss of endothelial MEF2C had no detected effects in physiological conditions, Xu et al. (2012) reported a significant increase in vascular recovery and a cognate decrease in pathological neovascularization in the *Mef2C*-null retinal vasculature after oxygen-induced retinopathy, indicating that loss of MEF2C results in increased sprouting after injury. These results, which correlate with work demonstrating reduced in vitro VEGFA-induced vascular sprouting after MEF2C overexpression (Sturtzel et al. 2014), are in agreement with the consequences of *Dll4* perturbations: Reduced DLL4 levels result in increased retinal vascular recovery and decreased neovascularizion after oxygen-induced retinopathy (Xu et al. 2012) and increased sprouting in response to VEGFA (Sainson et al. 2005; Jakobsson et al. 2010), while overexpression of *DLL4* reduces the in vitro responses to VEGFA (Williams et al. 2006). Therefore, we hypothesize that even limited reductions of *Dll4* levels downstream from MEF2C perturbation disrupt Notch-mediated lateral inhibition, resulting in hypersprouting in conditions of pathological stress.

Functional redundancy within the MEF2 family may not be the only contributor to the variable phenotypes seen after *Mef2* gene deletion. The multiple targets of MEF2 factors in the vasculature, particularly those involved in angiogenic sprouting, may also contribute to the challenges in detecting and understanding phenotypic changes after MEF2 factor modulation. The reduced vascular density, coverage, and tip cell numbers seen in iEC MEF2A/C retina vasculature would initially appear opposite to the hypersprouting seen after *Dll4* reduction, although reduced Dll4 expression was consistently seen in these mice. However, this phenotype must be considered in light of other roles for MEF2 factors in the vasculature. MEF2 has been implicated in the regulation of vascular integrity downstream from MMP10 activation and in the activation of the transcription factors KLF2 and KLF4 (Parmar et al. 2006; Maejima et al. 2014), important regulators of inflammation, vascular tone, and stabilization (Atkins and Jain 2007). Additionally, our work here demonstrates a direct link between MEF2 factor and many other genes involved in sprouting angiogenesis. The challenge in understanding the phenotypes after Mef2 ablation comes because individual loss of different genes required during sprouting angiogenesis can result in vastly different consequences. While depletion of *Dll4* levels can have a proangiogenic effect, loss or depletion of direct MEF2 targets ETS1, ELK3, and HLX can have anti-angiogenic effects in vivo (Pham et al. 2007; Wei et al. 2009; Herbert et al. 2012; Weinl et al. 2014). Furthermore, the vascular Notch pathway genes disregulated by DLL4 reduction are themselves, like all other vascular genes, likely to be direct ETS factor targets, suggesting that the hypersprouting routinely seen after Dll4 disruption may also be indirectly repressed by the general suppression of MEF2–ETS–Notch pathway gene activation. Consequently, while the balance of pro- and anti-angiogenic signaling appears perturbed only in MEF2C-null retina when pathologically stressed, the compound loss of MEF2A/C appears to tip the balance into an anti-angiogenic response, albeit one potentially mitigated by the maintained MEF2D signaling and possibly the disruption of Dll4-mediated lateral inhibition. These results also serve to illustrate the challenges of interpreting knockout animal models when studying transcription factors with multiple targets, particularly when redundancy must also be considered.

In conclusion, our work demonstrates that the analysis of enhancers can be a powerful approach to study the heterogeneity and complexity of signaling networks operating in angiogenesis in a variety of different settings.

## Materials and methods

### Cloning

The Dll4in3, Dll4in3mutMEF2, and Dll4-12 enhancers were as described previously (Sacilotto et al. 2013). The ETS1+194, ELK3-29, HLX-3, HLX-3mutMEF2, hlx-3, and hlx-3mutMEF enhancers were generated as custom-made, double-stranded linear DNA fragments (GeneArt Strings, Life Technologies). Reporter vectors were generated using Gateway technology (Invitrogen).

### Animals

All UK animal procedures were approved by local ethical review and licensed by the UK Home Office. US animal procedures complied with US federal and institutional guidelines. Transgenic mice were generated by oocyte microinjection. Mosaic transgenic zebrafish embryos were generated using the tol2 system (Kawakami 2005), and GFP reporter expression was scored at 32 hpf. The *tg(hlx-3:GFP)* stable line was created by outcross of adult F0 carriers generated using the tol2 system. X-gal analysis of embryos and postnatal organs was as described (De Val et al. 2004). X-gal analysis in retinas was as described (Jakobsson et al. 2010), and hindbrains were dissected as described (Fantin et al. 2013) and treated like the embryos. For Matrigel assays, transgenic mice were subcutaneously injected into the flanks with BD Matrigel basement membrane matrix (BD) supplemented with 2 µg/mL fibroblast growth factor (Peprotech) and harvested 14 d after injection. For tumors, transgenic mice were subcutaneously injected with 100 µL of BD Matrigel basement membrane matrix (BD) containing 1 × 10^5^ B16F10 melanoma cells and harvested at 12-mm diameter. iEC *Mef2A/C* knockout embryos and pups were obtained by crossing Mef2A^flox/flox^;Mef2C^flox/flox^ mice (Vong et al. 2005; Akhtar et al. 2012) with Cdh5(PAC)^Cre-ERT2^ mice (Wang et al. 2010). Recombination was induced by tamoxifen injection in pregnant mice 9 d after a plug was detected, and embryos were harvested 3 d later, genotyped, and fixed in 4% PFA for 2 h. For postnatal analysis, tamoxifen injection occurred at P1, P2, and P3, and retinas and lungs were harvested at P5 and P6. Eyes were removed from pups, fixed with 4% PFA for 90 min, and rinsed in PBS, and then the retinas were dissected as described previously (Pitulescu et al. 2010). Whole lungs were also dissected, rinsed in PBS, and then snap-frozen in liquid nitrogen for subsequent RNA extraction.

### Western blot

HUVEC pellets were lysed in RIPA buffer (50 mM Tris-HCL at pH 7.4, 1% NP-40, 0.5% Na-deoxycholate, 0.1% SDS, 150 mM NaCl, 2 mM EDTA) supplemented with protease and phosphatase inhibitor cocktail (Roche). Hindbrains were lysed in Tris buffer (20 mM Tris at pH 9, 2% SDS) supplemented with protease and phosphatase inhibitor cocktail (Roche), boiled at 100°C, and then incubated at 750 rpm at 80°C. Insoluble material was removed, protein concentration was determined with the BCA protein assay kit (Thermo Scientific), and 10 μg of protein per lane was separated by SDS-PAGE. DLL4 was detected using anti-DLL4 (1:1000; Abcam, 7280) and membranes were reprobed with anti-β-Actin antibody ( 1:100,000; Abcam, clone AC-15). Band intensities of DLL4 and β-Actin were quantified with ImageLab software (Bio-Rad).

### Immunostaining

Hindbrains and retinas were processed as described (del Toro et al. 2010; Pitulescu et al. 2010; Fantin et al. 2013), and tumors and E10 embryos were fixed in 4% PFA for 1 h on ice. After incubation in blocking solution (10% normal donkey serum, 0.1% [v/v] Triton X-100 in PBS), samples were incubated overnight at 4°C with the designated primary antibodies (MEF2A [Abcam], MEF2B [Abcam], MEF2C [Cell Signaling], MEF2D [BD], EP300 [Active Motif], HDAC4 [GeneTex], DLL4 [R&D Systems] isolectin B4 [IB4] [Vector Laboratories], and Erg [Abcam]) in 0.1% PBS-T. Samples were washed in PBS-T and incubated for 3 h with suitable species-specific Alexa fluor- or biotin-conjugated secondary antibodies in 0.1% PBS-T. Total numbers of branch points and tip cells and retinal outgrowth length were measured after IB4 staining using ImageJ software from pooled images of retinas from at least two independent litters.

### Cell culture and ChIP

HUVECS (pooled) (Lonza) were grown as described previously (Strasser et al. 2010; Sacilotto et al. 2013). For VEGF stimulation, cells were starved for 18 h in EBM-2 medium (Lonza) and then incubated for 1 h with EBM-2 supplemented with 25 ng/mL VEGFA_165_ (Peprotech, Lonza). ChIP assays were performed as described (Sacilotto et al. 2013) using antibodies against EP300 (Active Motif, 61401), HDAC4 (Proteintech, 60105-1), HDAC7 (Epigentek, A4007-050), HDAC9 (BioOrbyt, orb214926), Mef2A (Abcam, ab109420), Mef2C (Cell Signaling, 5030S), and Mef2D (BD, 610774). RNA isolation, cDNA synthesis, RT–PCR, and quantitative RT–PCR (qRT–PCR) were performed as described previously (Nikitenko et al. 2013) using primer/probes from Applied Biosystems. Dharmacon siRNA targeting human MEF2A, MEF2C, MEF2D, or the negative control sequence were purchased from GE Healthcare. Stealth siRNA targeting mouse Mef2A, Mef2C, and Mef2D were purchased from Life Technologies. See the Supplemental Material for sequences and catalog numbers. siRNAs were transfected into primary HUVECs or bEND cells expressing GFP (pGIPz-GFP-Puro HUVECs) at a final concentration of 100 nM using Oligofectamine (Life Technologies). MEF2A, MEF2C, or MEF2D stable knockdown was performed using lentiviral particles containing shRNAmir for MEF2A, MEF2C, MEF2D, or nonsilencing target (Open Biosystems).

### Bioinformatic analysis of MEF2-enriched binding sites

MEF2C and corresponding H3K27ac ChIP-seq data were obtained using publicly available ChIP-seq data (Maejima et al. 2014) accessible at NCBI Gene Expression Omnibus database (Edgar et al. 2002), accession numbers GSE32547, GSE32644, GSE32693, and GSE41553. Raw reads were trimmed with Sickle, and duplicate PCR reads were removed with rmdup. Reads were then aligned to human genome build hg19 using Bowtie2, and peaks were called with MACS2. For the enrichment analysis, RefSeq genes were obtained from University of California at Santa Cruz, and antisense genes were removed. MEF2C peaks were required to reside <500 base pairs from a H3K27ac peak. Fisher's exact was used to determine enrichment of MEF2C peaks in or around genes up-regulated in tip cells.

### HDAC inhibitor assays

HUVECS were starved for 16 h in EBM-2; treated with 400 nM TSA, 10 µM BML-210 (Abcam), 10 µM MC-1568 (Sigma), or DMSO (incubation times are in the figure legends); and stimulated with EBM-2 supplemented with 25 ng/mL VEGF-A for 1 h before harvesting for RNA extraction. Embryo culture in TSA was as described previously (Rojas et al. 2005) with modifications: Whole embryos (with their heads) were incubated for 17 h in either 100 µM TSA or control DMSO. In utero intracerebral injections were as described previously (Garcia-Moreno et al. 2014): Each E12 embryo was injected with ∼1 μL of 10 µM TSA or DMSO control, dyed with fast green into the fourth ventricle, left in utero for 24 h, and then harvested.

### Gene expression analysis

RNA extraction was carried out using the RNAspin minikit (GE Healthcare) following the manufacturer's instructions from either HUVECs (directly lysed on the cell culture plate in R1 buffer) or whole-lung extracts (homogenized in liquid nitrogen using a pestle and mortar and subsequently lysed in R1 buffer). Total RNA (0.5–1 µg) was then retrotranscribed using random primers and SuperScript III (Life Technologies) as per the manufacturer's instructions, and the cDNA was analyzed by qRT–PCR (StepOne Plus, Life Technologies) using gene-specific TaqMan probes (Applied Biosystems).

## Supplementary Material

Supplemental Material
