# Supplementary material for: MEF2 transcription factors are key regulators of sprouting angiogenesis
Source: Genes Dev. 2016 Oct 15;30(20):2297–309. doi: 10.1101/gad.290619.116 (PMC5110996; doi:10.1101/gad.290619.116)
Supplement: Supplemental Material [file supp_30_20_2297__index.html]

Supplemental Material 

# MEF2 transcription factors are key regulators of sprouting angiogenesis

## Supplemental Material

**Files in this Data Supplement:**

- Supplemental\_Figures.pdf
- Supplemental\_Material.pdf
- Supplemental\_Tables.pdf
