# Supplementary material for: MEF2 transcription factors are key regulators of sprouting angiogenesis
Source: Genes Dev. 2016 Oct 15;30(20):2297–309. doi: 10.1101/gad.290619.116 (PMC5110996; doi:10.1101/gad.290619.116)
Supplement: Supplemental Material [file supp_30.20.2297_Supplemental_Figures.pdf]

Supplemental Figure 1, related to Figure 1

(A) Endogenous DLL4 expression (green) in E10 embryo, E11 hindbrains and P6 retina. a artery, sa, sprouting angiogenesis.

(B) Representative images from Dll4in3:*LacZ* transgenic retina at post-natal day 5 (P5) demonstrate X-gal activity (blue and pseudocoloured green) in arteries (a) and at the angiogenic front (AF). X-gal expression was detected in most, but not all, endothelial cells at the tip-cell position (*). Expression is not detected in every endothelial cell as imaged through isolectin B4 (IB4) staining, including no expression in veins (v).

(C) Representative images from Dll4in3:*LacZ* transgenic retina from post-natal day 4 (P4) through P10, when angiogenic sprouting is complete. X-gal expression (black) is seen in arteries (a) and at the angiogenic front (AF), but excluded from veins (v). Whole vasculature (white) was detected by isolectin B4 (IB4).

(D) Expression patterns for Dll4in3 and Dll4-12 transgenes in E11 hindbrains from independent transgenic insertion events (trans, transient; line, stable line), n, neuronal staining. Similar vascular expression patterns were seen in all samples, although the extent of ectopic neural expression was variable, as is commonly seen for transgenes using hsp68 as a minimal promoter.

(E) Representative images of Dll4in3:*LacZ* and Dll4-12:LacZ transgene expression in hearts and peritoneum from the same animals from which the retina images in Figure 1 were obtained. Both transgenes direct robust expression in arterial endothelial cells (a), although Dll4in3:*LacZ* has a larger domain of expression.

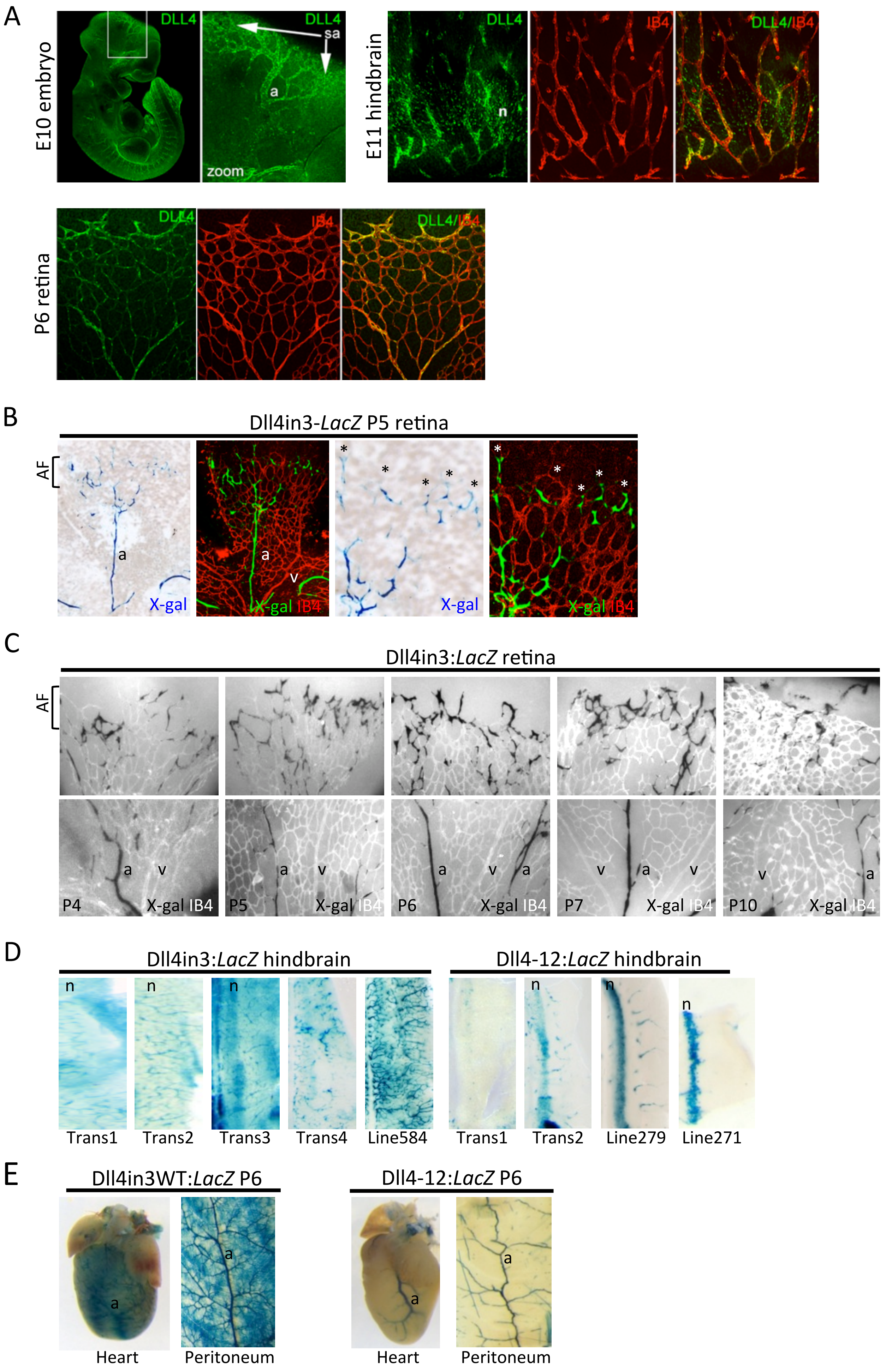
A
E10 embryo
DLL4
DLL4
sa
a
zoom
E11 hindbrain
DLL4
IB4
DLL4/IB4
n
P6 retina
DLL4
IB4
DLL4/IB4
B
Dll4in3-*LacZ* P5 retina
AF
a
X-gal
a
v
X-gal IB4
X-gal
X-gal IB4
C
Dll4in3:*LacZ* retina
AF
a v
P4 X-gal IB4
a v
P5 X-gal IB4
a v a
P6 X-gal IB4
v a v
P7 X-gal IB4
v a
P10 X-gal IB4
D
Dll4in3:*LacZ* hindbrain
Dll4-12:*LacZ* hindbrain
n
Trans1
Trans2
Trans3
Trans4
Line584
Trans1
Trans2
Line279
Line271
E
Dll4in3WT:*LacZ* P6
Dll4-12:*LacZ* P6
a
Heart
Peritoneum
Heart
Peritoneum

Supplemental Figure 2, related to Figure 2

(A-B) Full sequences of mouse Dll4in3 enhancer (A), aligned with orthologous zebrafish (zfish) sequence, and the mouse Dll4-12 enhancer (B), aligned with orthologous opossum sequence (opos) using the ClustalW program (Thompson et al., 1994), conserved base-pairs indicated with *. Verified transcription factor binding motifs are marked by coloured boxes, known consensus or near-consensus binding motifs that were experimentally verified but did not bind are marked by grey boxes.

(C-D) The Dll4in3 MEF2 motif robustly binds MEF2A, MEF2C and MEF2D proteins in EMSA analysis. (C) Radiolabeled oligonucleotide probe encompassing a control MEF2 binding site (Esser et al.) is bound by recombinant MEF2A, MEF2C and MEF2D protein (lanes 2, 5 and 8), and is competed by an excess of Dll4in3 enhancer MEF2 motif oligo (Dll4 MEF2 WT, lanes 3, 6 and 9), but not when this sequence contained a 5bp mutation within the MEF2 binding motif (Dll4 MEF2 MT, lanes 4, 7 and 10).

(D) Radiolabeled oligonucleotide probe encompassing the Dll4in3 MEF2 site is directly bound by recombinant MEF2A, MEF2C and MEF2D proteins (lanes 12, 15 and 18), and is competed by an excess of unlabeled self-probe (Dll4 MEF2 WT, lanes 13, 16 and 19) but not mutant self-probe (Dll4 MEF2 MT, lanes 14, 17 and 20).

(E) MEF2 factor binding at the DLL4in3 enhancer analysed by ChIP-qPCR after VEGFA stimulation in HUVECs.. Graph is representative of 4 biological replicates.

(F) Expression patterns for the Dll4in3mutMEF:*LacZ* transgene in E11 hindbrains from multiple transgenic insertion events (trans, transient; line,

stable line), n, neuronal staining. Similar vascular expression patterns were seen in all samples, although the extent of ectopic neural expression was variable, as is commonly seen for transgenes using hsp68 as a minimal promoter.

(G) Representative images of Dll4in3mutMEF:*LacZ* transgene expression in hearts and peritoneum from the same animals from which the retina images in Figure 2 were obtained. a, artery.

Sacilotto Supplementary Figure 2

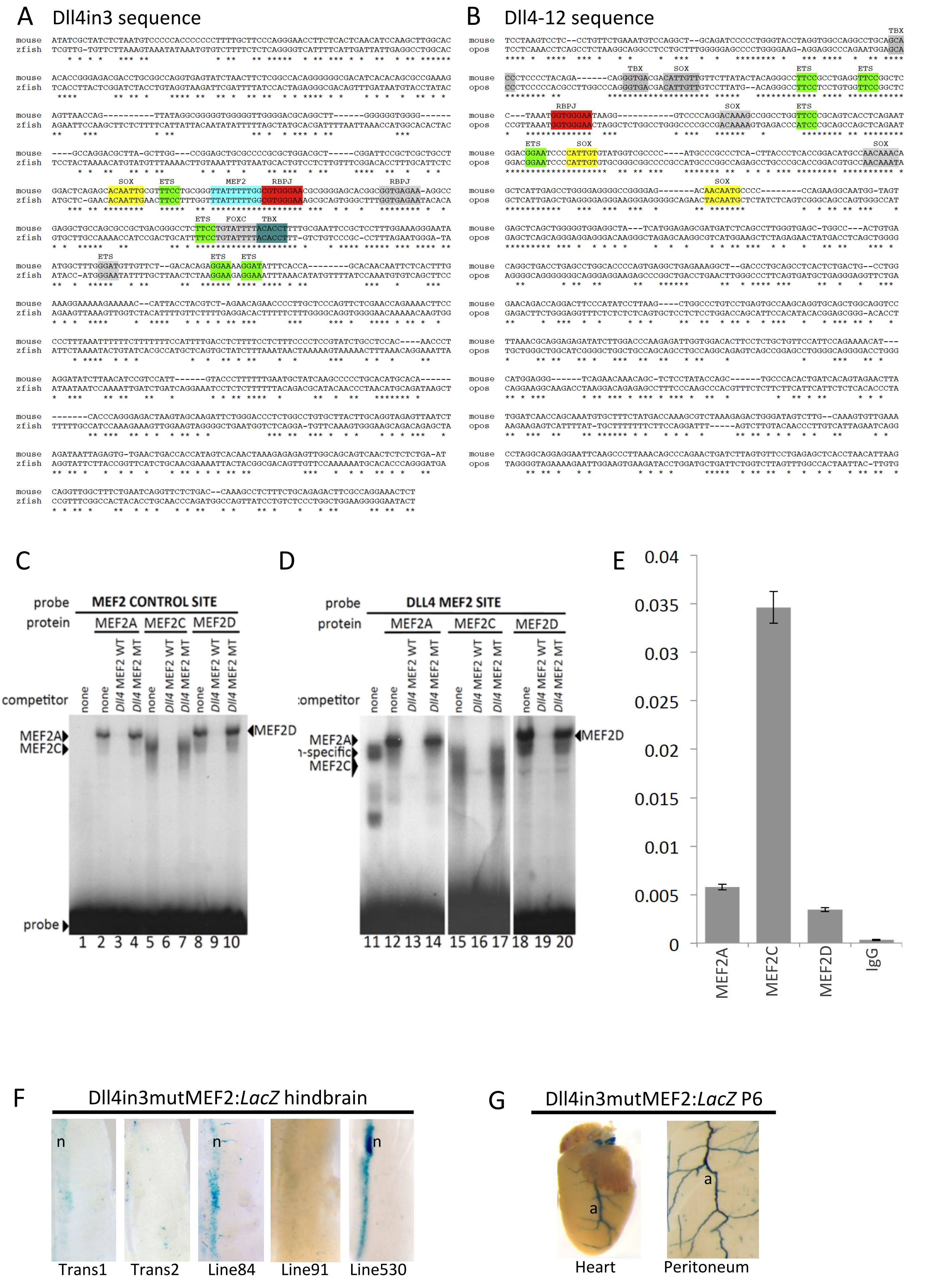

Supplemental Figure 3, related to Figure 3

(A) Immunostaining on sectioned X-gal-stained Matrigel plugs for the pan-endothelial marker CD31 (red). Matrigel plugs grown in Dll4in3WT:*LacZ* transgenic mice expressed X-gal in a subset of endothelial cells, whereas Matrigel plugs grown in Dll4in3mutMEF2:*LacZ* transgenic mice had no X-gal staining yet robust CD31 staining, indicating that the transgene was not expressed during neo-vascular growth into the Matrigel plug.

(B-D) Four representative Matrigel plugs and four representative B16F10 melanoma tumours grown in Dll4in3WT:*LacZ* transgenic mice (B) and Dll4in3mutMEF2:*LacZ* transgenic mice (C) and stained with X-gal demonstrate the typical variation in staining among experiments. Mean blue blood vessels per 100µm analysed is displayed in (D), N=4, error bars indicate standard error of the mean. Each transgenic mouse (all male) was functionally verified to ensure transgene activity by crossing with a WT female and analysis of E11 embryos. The bladder was also removed from each mouse (E) concurrent with matrix/tumour removal, and stained to verify that each animal model was genetically and functionally identical to others of the same line.

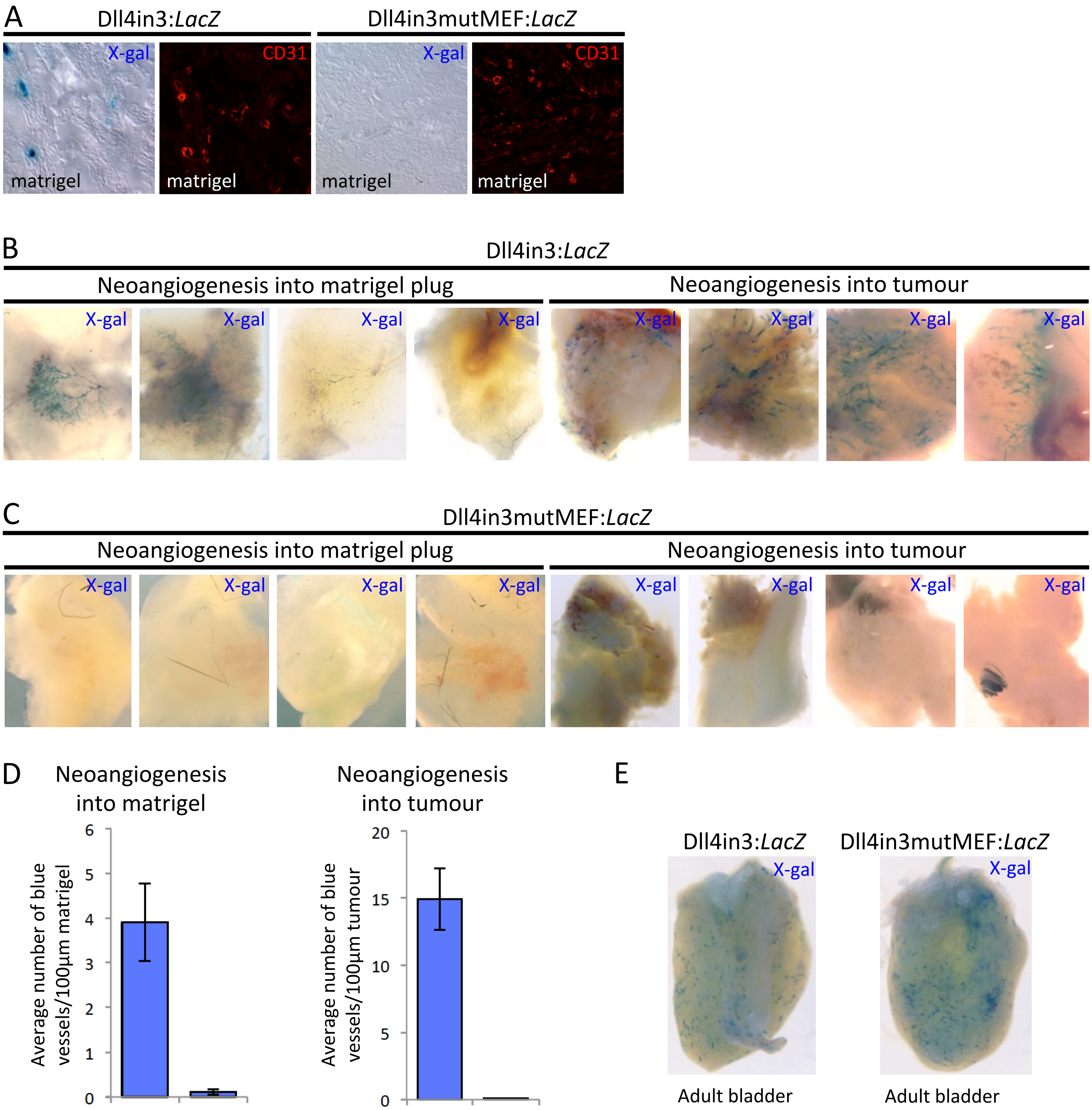

A
Dll4in3:*LacZ*
Dll4in3mutMEF:*LacZ*
X-gal
CD31
matrigel
B
Dll4in3:*LacZ*
Neoangiogenesis into matrigel plug
Neoangiogenesis into tumour
X-gal
C
Dll4in3mutMEF:*LacZ*
Neoangiogenesis into matrigel plug
Neoangiogenesis into tumour
X-gal
D
Neoangiogenesis into matrigel
Average number of blue vessels/100µm matrigel
0
1
2
3
4
5
6
Neoangiogenesis into tumour
Average number of blue vessels/100µm tumour
0
5
10
15
20
E
Dll4in3:*LacZ*
Dll4in3mutMEF:*LacZ*
X-gal
Adult bladder
Adult bladder

Supplemental Figure 4, related to Figure 4

(A) MEF2A, MEF2C are expressed in endothelial cells in the P5 mouse retina, and MEF2A, MEF2C and MEF2D are expressed in B16F10 melanoma subcutaneous tumours and human renal tumour, as detected by immunofluorescence. CD31 and isolectin B4 (IB4) label all endothelial cells.

(B) Specific shRNA knock-down of MEF2A, C and D in HUVECs affects the designated MEF2 factor only, as detected by immunofluorescence, and confirms that the MEF2A, C and D antibodies used do not cross-react significantly with other MEF2 family members.

(C) Time-course of VEGFR2, ERK1/2 and AKT phosphorylation after VEGFA stimulation in HUVECs, analyzed by western blot. Total levels of VEGFR2, ERK1/2 and AKT remain constant after stimulation whereas changes in pERK and pAKT indicate successful VEGFA stimulation.

(D) qRT-PCR analysis demonstrates increased expression of MEF2A, MEF2C, MEF2D and DLL4 in HUVECs 0-8 hours after VEGFA stimulation. Error bars indicate standard error of the mean of two biological replicates.

(E) Individual siRNA knock-down efficiently and specifically ablates the designated MEF2 factor in both mouse (bEnd3) and human (HUVECs) endothelial cells. MEF2A, C and D antibodies specifically recognize the designated family member in both mouse and human cell extracts.

(F) Relative *DLL4* expression in siControl-transfected HUVECs analysed by qRT-PCR before and after VEGFA stimulation. Directly comparable with data in Figure 4D. Graph is representative of 2 biological replicates.

Sacilotto Supplemental Figure 4

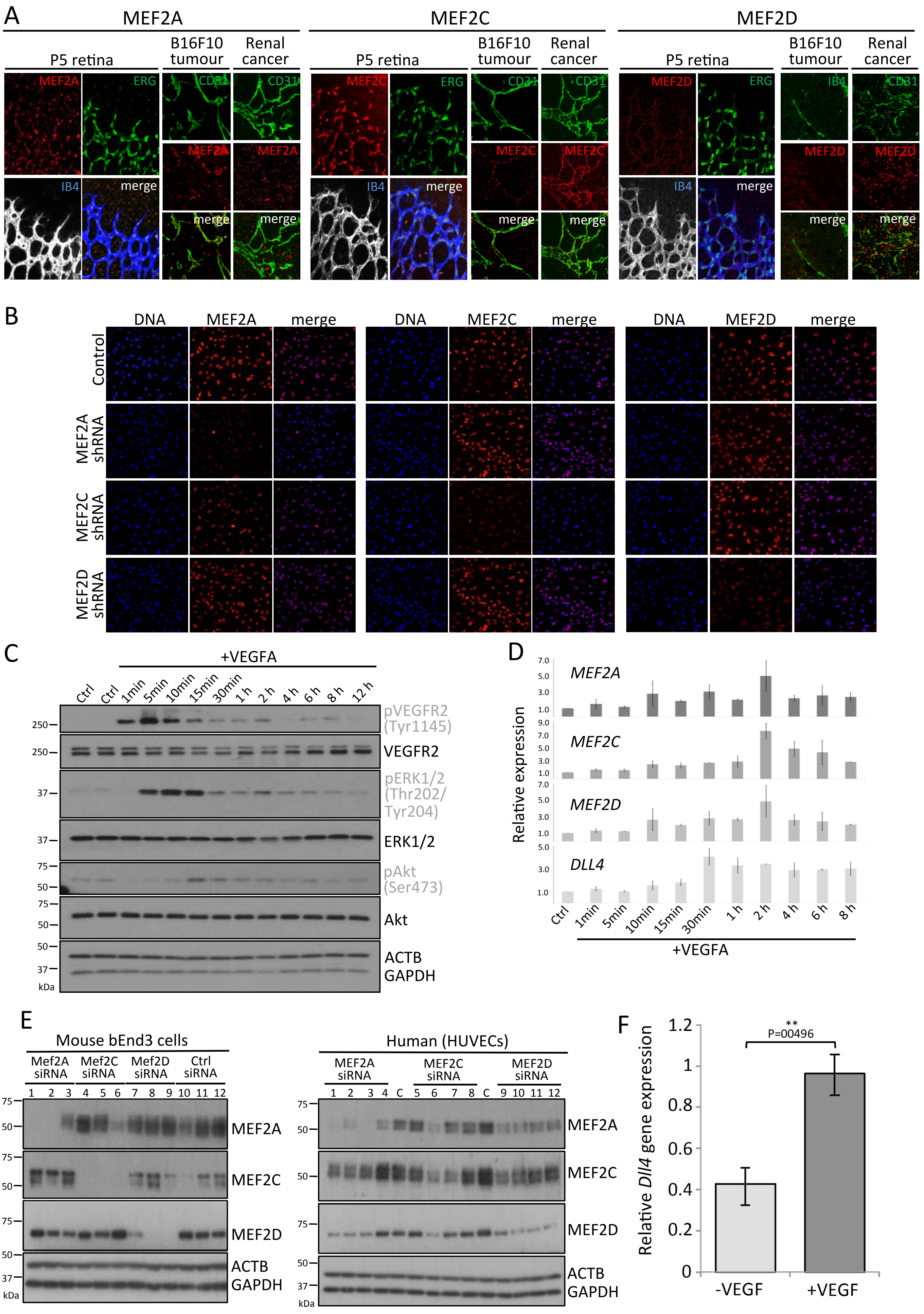

Supplemental Figure 5, related to Figure 4

(A-B) A representative image from chimeric HUVEC competition assay of wild-type (WT, mCherry-expressing; red) and combined MEF2A/C/D siRNA knock-down (KD, GFP-expression; green) cells mixed at a 1:1 ratio. WT cells are predominantly found at the tip cell position (indicated by red arrowhead) than MEF2A/C/D knock-down cells (indicated by green arrowhead). Quantification of tip cells (B, pooled images from three biological replicates) shows a significant reduction of MEF2A/C/D KD cells at the tip cell position. P value= 3.40e-08. Scale bars correspond to 200 μm.

(C-D) A representative picture from embryoid body competition assay of wild-type (WT, green) and CRISPR/Cas9-mediated MEF2A/C null ES cells (red), mixed at a 1:1 ratio. WT cells are more often found at the tip cell position (indicated by green arrowheads) than ΔMEF2A/C cells (indicated by red arrowheads). Quantification of WT and ΔMEF2A/C tip cells (D, using pooled images from two biological replicates) shows a significant decrease of ΔMEF2A/C cells at the tip cell position. P value= 2.20e-04. Four different CRISPR/Cas9-mediated mutant ES cell clones were used for this work, in each case the indel was confirmed by Sanger sequencing.

(E) Schematic detailing the creation of CRISPR/Cas9-mediated MEF2A/C null ES cells. Protein schematics of MEF2C and MEF2A adapted from Lin et al. 1997 and Naya et al. 2002. gRNAs were designed to target the portion of the MEF2 domain indicated in sequences (WT part. MEF2 domain). Deletions for ES cells shown in C are indicated in allele sequences and are representative for the four different ES clones.

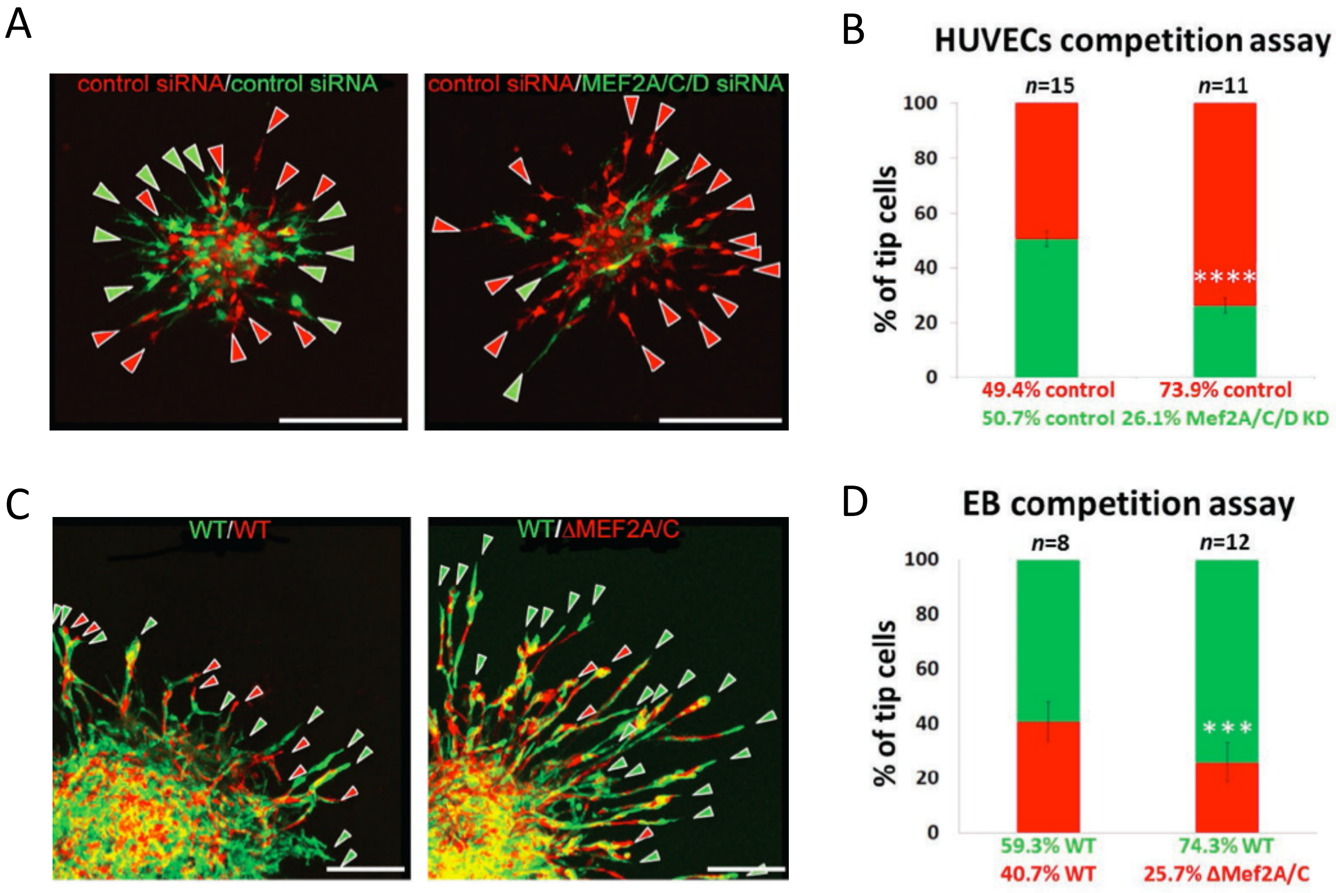

E

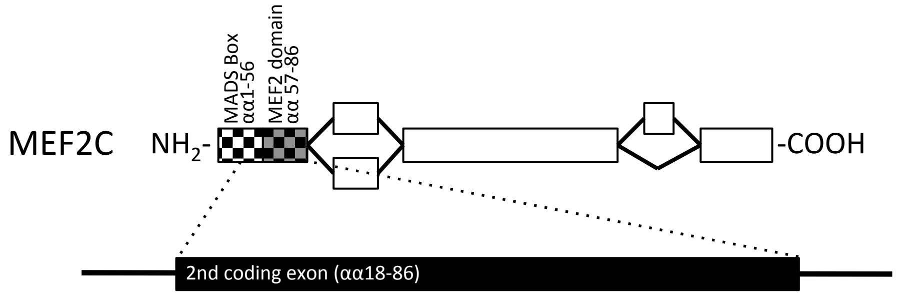

gRNA sequence	GAGTTTGTCCGGCTCTCGTG
WT 3’ part of MEF2 domain nt	AAGTACACCGAGTACAACGAGCCGCACGAGAGCCGGACAAACTCAGACATTGTGGAG
WT C terminal of MEF2 domain αα	K Y T E Y N E P H E S R T N S D I V E
Mef2c allele 1 seq	AAGTACACCGAGTACAACGAGCCGCAC-----------AAACTCAGACATTGTGGAG
Mef2c allele 2 seq	247bp deletion from αα22 in MADS box and entire MEF2 domain

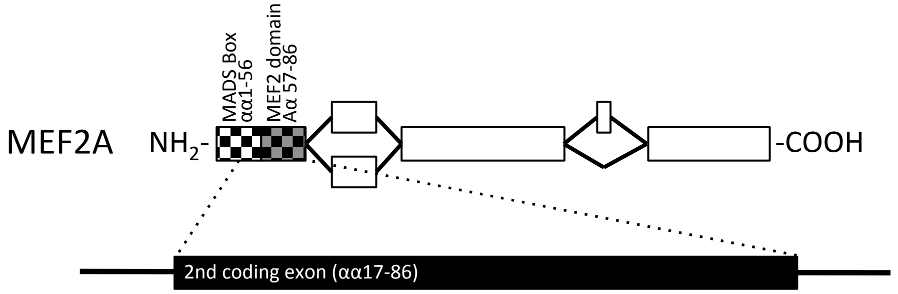

gRNA sequence	GAGTTCGTCCTGCTTTCATG
WT 3’ part of MEF2 domain nt	AAATACACTGAGTATAACGAGCCTCATGAAAGCAGGACGAACTCGGATATCGTTGAG
WT C terminal of MEF2 domain αα	K Y T E Y N E P H E S R T N S D I V E
Mef2a allele 1 seq	AAATACACTGAG-----------------------GACGAACTCGGATATCGTTGAG
Mef2a allele 2 seq	AAATACACTGAGTATAACGAG-----------CAGGACGAACTCGGATATCGTTGAG

Supplemental Figure 6, related to Figure 6

(A) MEF2C binding profile around the *DLL4* locus. Red box indicates statistically significant MEF2C binding region, red peaks indicate MACS2 bedgraph MEF2C peaks visualized in IGV, green lines indicate statistically significant H3K27Ac regions, and black lines indicate locations of previously tested orthologous mouse Dll4 enhancers.

(B) MEF2C binding peaks are enriched around 50kb of genes associated with sprouting angiogenesis, as assessed by increased expression in the hyper-sprouting retina of Dll4+/- mice (del Toro et al., 2010) (blue), or identified in retinal tip cells isolated through laser capture microdissection (Strasser et al., 2010) (pink).

(C) Genomic snapshots denoting MEF2C binding sites within the loci for Notch pathway genes. H3K27Ac peaks indicated in green, MEF2C binding peaks in red. Only one MEF2 binding peak was detected, around the Dll1 locus, but this was not co-localised within or around a H3K27Ac peak, a pre-requirment in our genomic MEF2 analysis.

Sacilotto Supplemental Figure 6

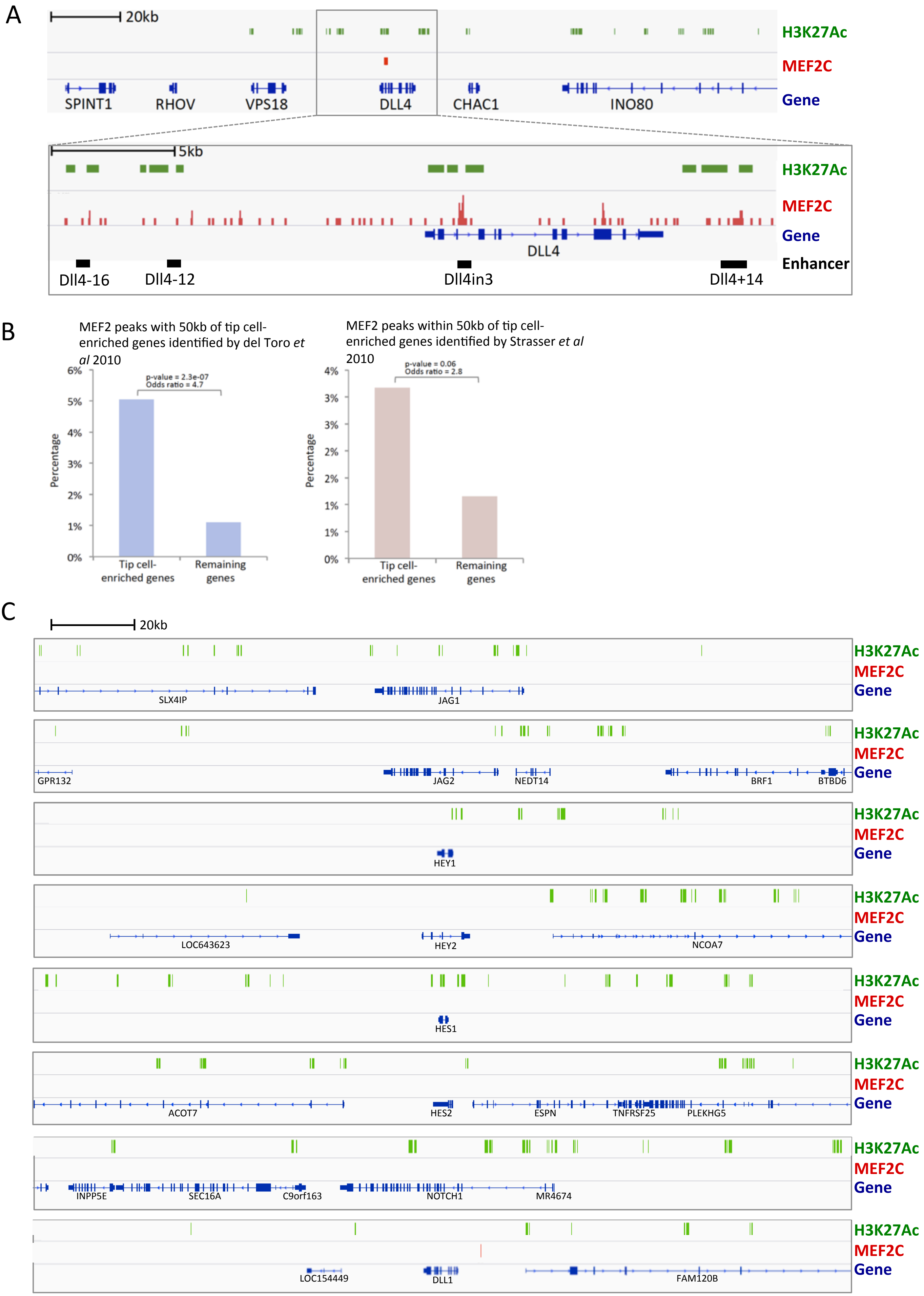

Supplemental Figure 7, related to Figure 6.

(A) MEF2C binding profile around the HLX locus. Red peaks indicate MACS2 bedgraph MEF2C peaks visualized in IGV, green lines indicate statistically significant H3K27Ac regions, black lines indicate locations of HLX-3 enhancer, grey line indicates region dynamically bound by EP300 after VEGFA stimulation (Zhang et al., 2013).

(B) Sequences of human HLX-3 and zebrafish hlx-3 enhancers aligned using the ClustalW program (Thompson et al., 1994), conserved base-pairs indicated with *. Verified transcription factor binding motifs are marked by coloured boxes, known consensus or near-consensus binding motifs that were not experimentally verified are marked by grey boxes.

(C) Representative 32hpf zebrafish embryo transgenic for the hlx-3mutMEF:GFP transgene. *denote ectopic expression in skeletal muscle fibres.

(D) Summary of reporter gene expression detected in E12 mice transgenic for the HLX-3WT:*LacZ* and HLX-3mutMEF:*LacZ* transgenes. * denotes transgenic mouse that expressed *LacZ* throughout embryo in all tissues, including all endothelial cells.

A

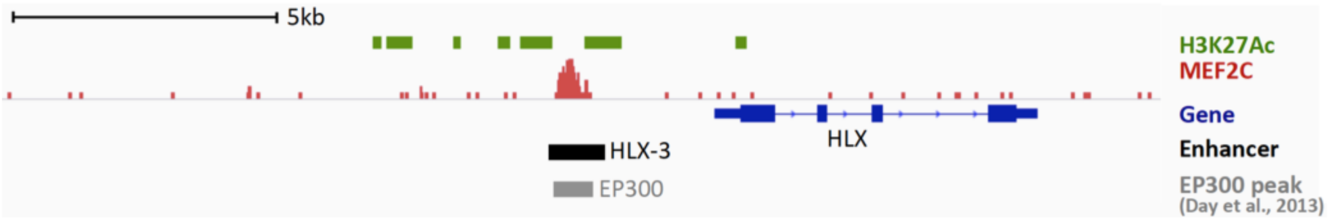

B

```
                             MEF2-a                           ETS-a      ETS-b                                             ETS-c
human   GTCGCAGCTGTATATTTATATTTTCATTGCTAGAAGGGAATTGATTTCCGTGCATTTATTTTGGTAGTTGTAATACATAAGGGCGGATTTGCGTCACCCGAGCAACTTGCCGGT
mouse   GTCGCAGCTGTGTATTTATATTTTCATTGCTAGAAGGGAATTGATTTCCGTGCATTTATTTTGGAAGTTGTAATACAGAAGAGCGGATTTGCGTCACCACAGCAACTTGCCGGT
zfish   GCCTCAGTAGTTAATTGTGTTTTCGTTTCAGC--GGAGAATAGATTTCCTCTGTTT-----ATTATAA-GCAT-----AAGGACGGATTTGCGTCACCGT-GCGACTTGCGAGT
        * * ***  *   ***    ***   **      * **** *******     **          * *     ***  ***************   ** *****    **
                                         ETS-d                                             ETS-e        ETS-f               MEF2-b
human   GGAGATAAAGTTGCACAAATATTGAAAGG-GGAAGTGCTAGGAGTCATTATAGAGTTTTTCTCC---------GGAAGAAATAAGGAT-TTCTGCAGTATCCTAAAATACTAAGG
mouse   GGAGATAAAGTTGCACAAATATTGAAAGG-GGAAGTGCTAGGAGTCATTATAGAGTTTTTCTCC---------GGAAGAAATAAGGAT-TTCTGCAGTATCCTAAAATACTAGGA
zfish   TGAGATAAAGTTGCACAAATATTGAAAAAAGGAAGTGCTAACAGTCATTATAAAGTTCCACCTCTGTGAGTCGGAGGAAATAAGGGTGCTCTGCTGTATCCTAAAATACTAAAG
        **************************   ********************** ****                  *** ********* *   ***** *****************
                MEF2-c                  ETS-g                                                                     ETS-h
human   CCGCTTCTATTTTGAG-ACCAATCTCGCAGGCACATCCGCTCATTTAGTCCCGAGTTTGAGCCCATCAAAAAA-CAGGAGATGACCTGAACTCCGG-CGAGCCCAGGGTTTCCTGC
mouse   CCGCTTCTATTTTGAG-ACCAATCTCGCAGGCATATCCGCTCATTTAG-CCCAAGTTTGAGCCCATCAAAAAAACAGGAGATGACCTGAACTCCGGGCGAGCCCCGGGTTTCCTGC
zfish   CATCTTCTATTTTTGGCCTAAATCTTGCAGGAATATCCGCTCATTTAG-CCTGAATCAGAGCTCATCAAAAA--CAGG--ATGACCTG--CATCGA--GAAACTCCTGTTTCCTAA
        *  **********  *    ***** ***** * ***************  *  * *  **** *********  ****  ********  *   **   **        *******
```

C

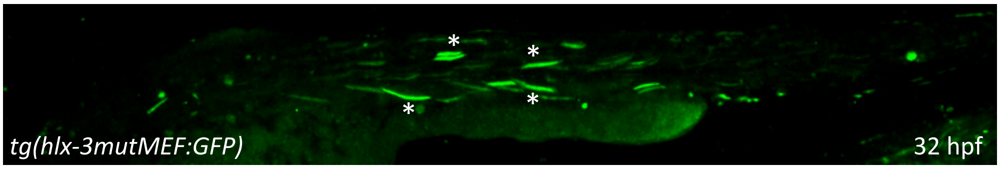

D

Transgene	Number of tg mice	Tg mice with any detectable X-gal expression	Tg mice with X-gal expression in angiogenic endothelial cells
*hlx-3*WT:*LacZ*	**11**	**9**	**7**
*hlx-3*mutMEF:*LacZ*	**10**	**10**	**0***

Supplemental Figure 8, related to Figure 6.

(A) The human HLX-3 and zebrafish hlx-3 enhancers robustly bind the ETS factors ETS1 (DNA binding domain DBD only) and ETV2 in EMSA analysis. Radiolabeled oligonucleotide probe encompassing the human sequence of five ETS binding motifs (ETS-b, d, e, g and h) were bound by recombinant ETS1DBD protein (lanes 2, 6, 10, 14 and 18), were competed by an excess of self-probe (lanes 3, 7, 11, 15 and 19), but not by mutant self-probe (lanes 4, 8, 12, 16 and 20). Radiolabeled oligonucleotide probe encompassing the zebrafish sequence of five ETS binding motifs (ETS-b, d, e, g and h) were also bound by recombinant ETV2 protein (lanes 22, 26, 30, 34 and 38), were competed by an excess of self-probe (lanes 23, 27, 31, 35 and 39), but not by mutant self-probe (lanes 24, 28, 32, 36 and 40).

(B) Radiolabeled oligonucleotide probe encompassing the human sequence of the three HLX MEF2 site were directly bound by recombinant MEF2C protein (lanes 2, 7 and 12), were competed by an excess of unlabeled self-probe (lanes 3, 8 and 13) but not mutant self-probe (lanes 4, 9 and 14). Radiolabeled oligonucleotide probe encompassing the orthologous zebrafish sequence of the three hlx MEF2 site were directly bound by recombinant MEF2C protein (lanes 16, 21, 26), were competed by an excess of unlabeled self-probe (lanes 17, 22 and 27) but not mutant self-probe (lanes 18, 23 and 28).

(C) Radiolabeled oligonucleotide probe encompassing the Dll4 MEF2 site, HLX MEF-c and *hlx* MEF-c sites were able to bind 2μl and 4μl MEF2A (lanes 1-12), MEF2C (lanes 13-24) and MEF2D (lanes 25-36) proteins at higher

affinity than control MLC MEF2 site. Dll4 MEF2 and HLX MEF2 sites were the strongest binders. All probes were used at 40,000 counts/minute.

Sacilotto Supplemental Figure 8

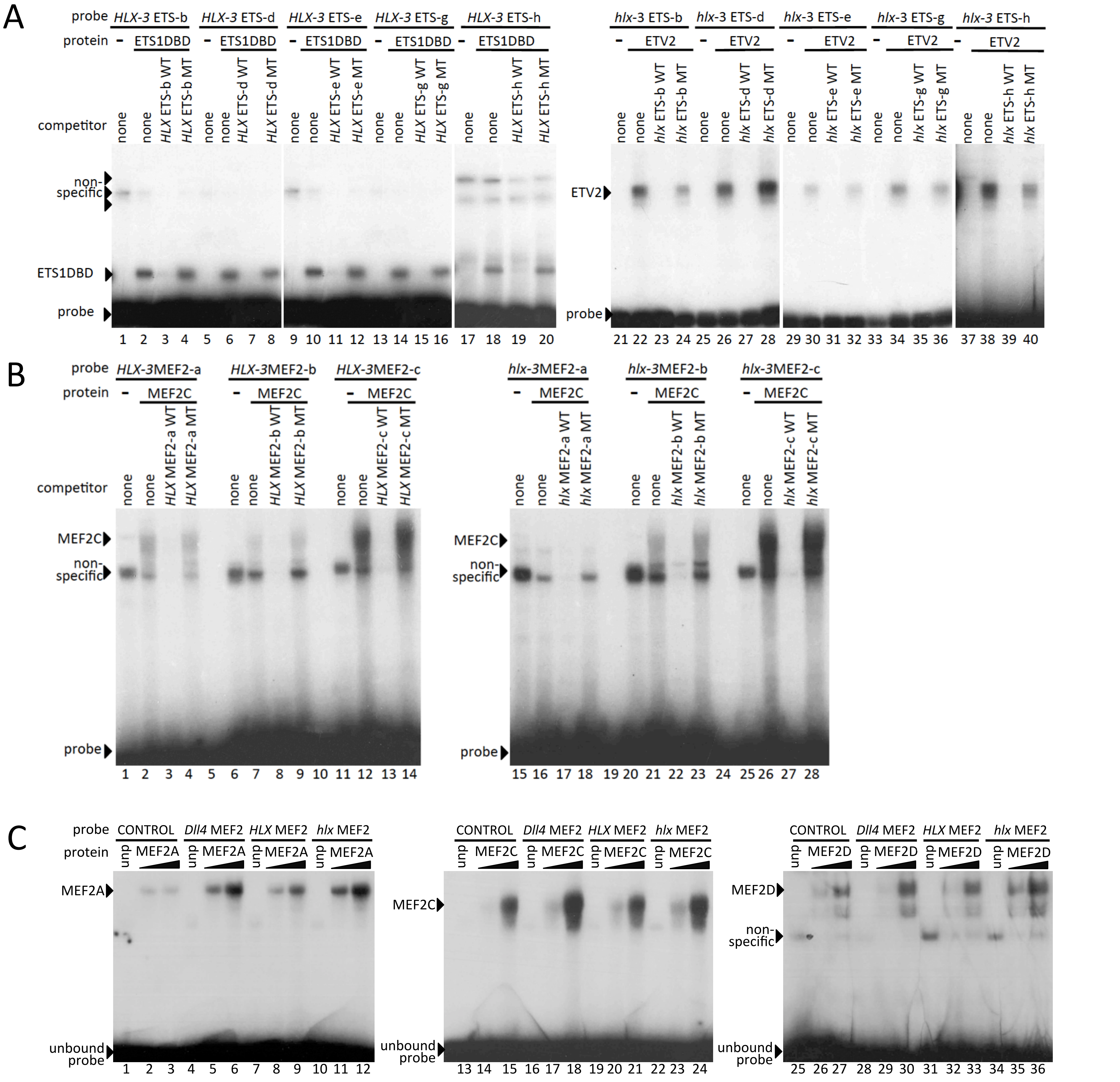

Supplemental Figure 9, related to Figure 7

(A) MEF2 factor binding at the DLL4in3 enhancer analysed by ChIP-qPCR before and after VEGFA stimulation in HUVECs. Graph is representative of 3 biological replicates.

(B) MEF2 factor binding at the HLX-3 enhancer analysed by ChIP-qPCR before and after VEGFA stimulation in HUVECs. Graph is representative of 3 biological replicates.

(C) Representative Dll4in3:*LacZ* embryos after 17 hours *ex vivo* incubation in medium +100μM TSA or DMSO followed by X-gal staining. TSA treatment resulted in expanded and ectopic expression of the transgene in Dll4in3:*LacZ* embryos, whereas less staining was detected in control DMSO-treated Dll4in3:*LacZ* embryos.

(D) Gene expression levels analysed by qRT-PCR in HUVECs treated with TSA and small molecule classII HDAC inhibitors BML-210 and MC-1568, after VEGF stimulation and relative to DMSO control. N=3. Genes shown are previously reported to be up- or down-regulated by TSA in the presence of VEGF but have no MEF2 binding motifs within 200kb.

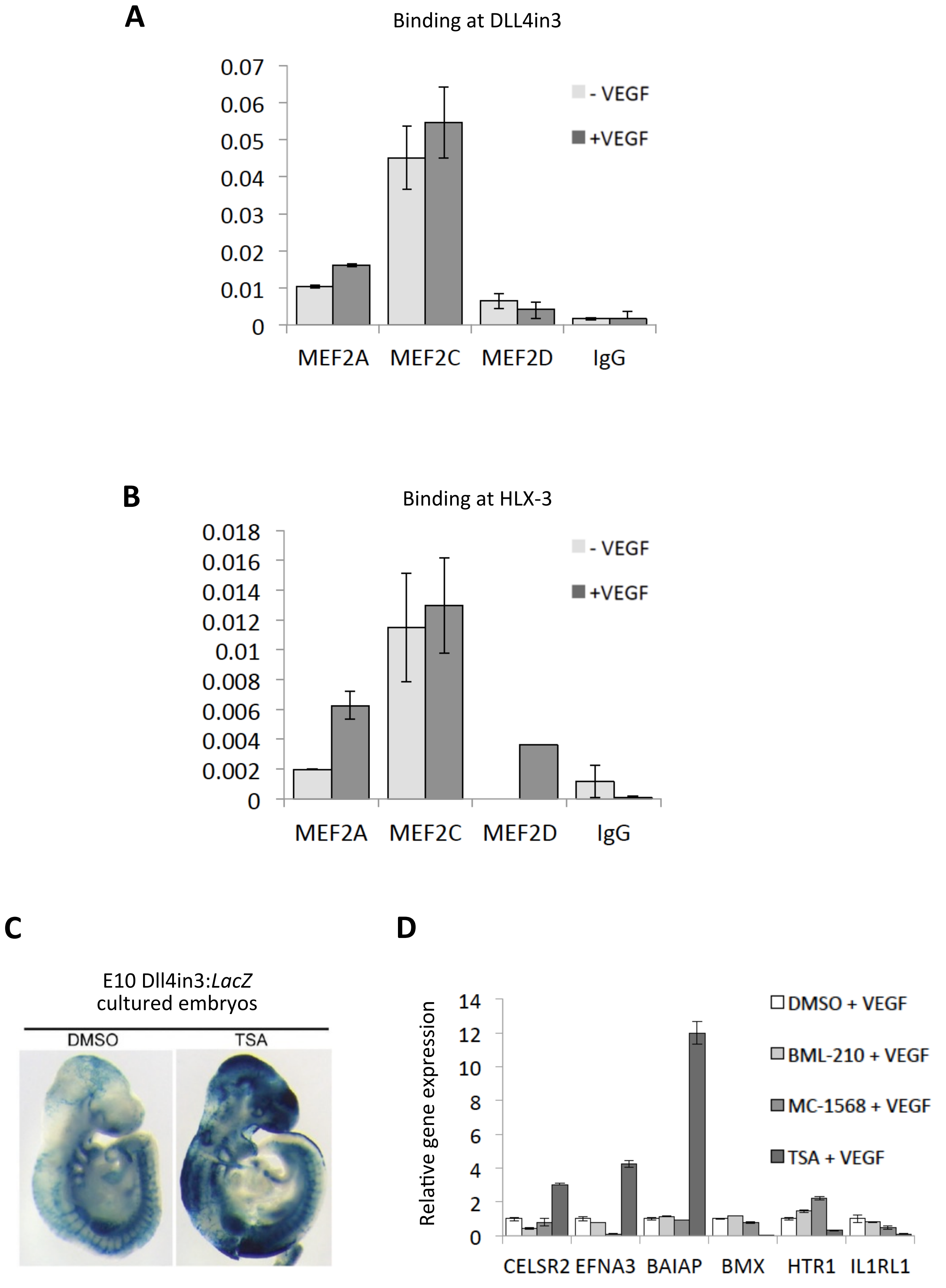
A
Binding at DLL4in3
0.07
0.06
0.05
0.04
0.03
0.02
0.01
0
- VEGF
+VEGF
MEF2A
MEF2C
MEF2D
IgG
B
Binding at HLX-3
0.018
0.016
0.014
0.012
0.01
0.008
0.006
0.004
0.002
0
- VEGF
+VEGF
MEF2A
MEF2C
MEF2D
IgG
C
E10 Dll4in3:*LacZ*
cultured embryos
DMSO
TSA
D
Relative gene expression
14
12
10
8
6
4
2
0
DMSO + VEGF
BML-210 + VEGF
MC-1568 + VEGF
TSA + VEGF
CELSR2
EFNA3
BAIAP
BMX
HTR1
IL1RL1
