# Supplementary material for: MEF2 transcription factors are key regulators of sprouting angiogenesis
Source: Genes Dev. 2016 Oct 15;30(20):2297–309. doi: 10.1101/gad.290619.116 (PMC5110996; doi:10.1101/gad.290619.116)
Supplement: Supplemental Material [file supp_30.20.2297_Supplemental_Material.pdf]

Supplemental Experimental procedures

Mice

All animal experiments were approved by a local ethical review committee and licensed by the UK Home Office.

Cloning of enhancers constructs

The Dll4in3, Dll4in3mutMEF2 and Dll4-12 enhancers were made as previously described (Sacilotto et al. 2013). The ETS1+194, ELK3-29, HLX-3, HLX-3mutMEF2, *hlx-3* and *hlx-3*mutMEF enhancers were generated as custom-made, double-stranded linear DNA fragments (GeneArt® Strings™, Life Technologies). These were cloned into the pCR8 vector using the pCR8/GW/TOPO TA Cloning Kit (Invitrogen, K2500-20) following manufacturer's instructions. The sequences of each enhancer DNA fragment are provided in the DNA sequence information below. Once cloning was confirmed, the enhancer sequence was transferred from the pCR8/GW/enhancer entry vector to a suitable destination vector using Gateway LR Clonase II Enzyme mix (Life Technologies, 11791-100) following manufacturer's instructions. For mouse transgenesis, the enhancer was cloned into the hsp68-LacZ-Gateway vector (provided by N. Ahituv). For zebrafish transgenesis, the enhancer was cloned into the E1b-GFP-Tol2 vector (provided by N. Ahituv).

Generation and analysis of transgenic mice

Transgenes were digested and gel purified (Qiagen) from the plasmid backbone following manufacturer's instructions, then re-suspended in water at a concentration of 2 ng/µl. Transgenic mice were generated by oocyte microinjection as described previously (De Val et al. 2004). Transgenic embryos or post-natal tissue samples were collected at indicated time points for transient analysis or were allowed to

develop to adulthood for establishment of stable transgenic lines. For hindbrain analysis, E11-E12 embryos were dissected and hindbrains removed prior to fixation as previously described (Fantin et al. 2013). Matrigel and xenograft samples were dissected away from other tissues prior to fixation. β-galactosidase expression was detected by X-gal staining: embryos/tissue were fixed in 2% PFA, 0.2% glutaraldehyde in PBS at 4 °C for 40 min to two hours, depending of the age of the embryo/size of tissue. Embryos were rinsed twice in PBS and stained for β-galactosidase in X-gal staining solution (5 mM $K_4Fe(CN)_6$, 5 mM $K_3Fe(CN)_6$, 2 mM $MgCl_2$, 0.2% Nonidet P40, 2.4 mM sodium deoxycholate, 1 mg/ml 5-bromo-4-chloro-3-indolyl β-d-galactopyranoside (X-gal), in PBS) over-night at room temperature. Imaging of whole embryos was performed using a stereo microscope (Leica M165C) equipped with a ProGres CF Scan camera (and ProgRes CapturePro software, Jenoptik). For histological analysis to investigate X-gal staining patterns, embryos/tissue samples were dehydrated through a series of ethanol washes, cleared by xylene and paraffin wax-embedded. 5 or 6-µm sections were prepared, de-waxed, and counterstained with nuclear fast red (Electron Microscopy Sciences). To image the vasculature in hindbrains, X-gal stained hindbrains were fixed in 4% PFA overnight, washed in 0.05% PBS-Triton X100 and stained with 1:100 DyLight-594 Isolectin B4 (Vector Laboratories) in 0.05% PBS-Triton X100, over-night at 4°C in the dark. Hindbrains were rinsed in 0.05% PBS-Triton X100 for 10 minutes and mounted on a microscope slide with a central cavity (Marienfeld) with Vectashield antifade mounting medium with DAPI (Vector Laboratories) for imaging with the Zeiss 710MP confocal microscope.

For reporter analysis in post-natal retinas, eyes were enucleated from transgenic pups at the indicated time-points, fixed in 4% PFA for 1 hour on ice, dissected in ice-cold PBS, washed twice in detergent rinse (0.1 M Phosphate buffer (pH 7.3), 2mM $MgCl_{2,}$ 0.01% sodium deoxycholate, 0.02% Nonidet P-40) for 20 mins and stained in retinal X-gal solution as previously described (Jakobsson et al. 2010) (0.1M Phosphate buffer (pH 7.3), 2mM $MgCl_{2,}$ 0.01% sodium deoxycholate, 0.02% Nonidet P-40, 5mM potassium ferricyanide, 5mM potassium ferrocyanide, 1 mg/ml 5-bromo-4-chloro-3-indolyl β-d-galactopyranoside (X-gal)) over-night at 37°C. After X-gal staining, retinas were post-fixed in 4% PFA over-night at 4°C and stained with 1:100 DyLight-594 Isolectin B4 (Vector Laboratories) in 0.05% PBS-Triton X100, over-night at 4°C. Retinas were washed in 0.05% PBS-Triton X100 and flat-mounted on a microscope slide with Vectashield antifade mounting medium with DAPI (Vector Laboratories) for imaging with the Zeiss 710MP confocal microscope.

For genotyping, ear biopsies from F0 mice were digested in 100 µl of GNT buffer (50mM KCl, 1.5mM $MgCl_2$, 10mM Tris Ph 8.5, 0.01% gelatin, 0.45% Nonidet P40, 0.45% Tween 20) with 2 µl 10 mg/ml proteinase K (Fisher Scientific) over-night at 55°C, and 0.5-2 µl were directly used for LacZ genotyping by PCR. LacZ positive adult mice were then crossed with WT mice and their progeny (embryos) tested for transgene expression by X-gal staining. Transgenic males producing X-gal positive progeny were selected as founders to establish stable transgenic lines.

For endothelial-specific inducible deletion of Mef2A and Mef2C, we crossed the Mef2Aflox/flox;Mef2Cflox/flox mice with Cdh5(PAC)-Cre-ERT2 mice (Wang et al. 2010), to produce iECKO Mef2A/C mice. For E12 hindbrain analysis, recombination

was induced with a single IP injection of 1 mg tamoxifen and 0.5 mg of progesterone in 0.1 ml solution in pregnant mice, nine days after a plug was detected. The drugs were diluted in ethanol and sunflower oil to make a solution of 10mg/ml tamoxifen and 5 mg/ml of progesterone with a final percentage of ethanol of 5%. Embryos were harvested two days later, genotyped and fixed in 4% PFA for two hours, after which the hindbrains were dissected as previously described (Fantin et al. 2013). For post-natal retina and lung analysis, intragastric injections of 50µl of tamoxifen solution at 1mg/ml (prepared in sunflower-seed oil containing 2.5% Ethanol) were performed once every 24 hours at P1, P2 and P3 as previously described (Pitulescu et al. 2010). Eyeballs were removed from pups, fixed in 4% PFA for 90 minutes, then rinsed in PBS and stored at 4°C until needed. The retina was then dissected as previous described (Pitulescu et al. 2010), and then immunostained for DLL4 and IB4 as described elsewhere. Lungs from the respective pups were also harvested, snap-frozen in liquid nitrogen and stored at -80°C until use. RNA extraction from lung extracts obtained by disruption of tissue in nitrogen-cooled mortar and pestle, was carried out using the Illustra RNAspin mini kit (GE Healthcare) following manufacturer's instructions and used for retro-transcription (Superscript III, Life Technologies) and qPCR (StepOne Plus, Life Technologies) using pre-designed Taqman assays (Applied Biosystems).

iECKO Mef2A/C and control hindbrains were lysed in Tris buffer (20mM Tris pH9, 2% SDS, supplemented with protease and phosphatase inhibitor cocktail, Roche), boiled at 100°C for 20 minutes followed by a 2 hour incubation at 80°C at 750 rpm. Insoluble material was pelleted at 14000 rpm for 20 minutes. Protein concentration was determined with the BCA Protein Assay kit (Thermo Scientific) and 10µg of

protein per lane was separated by SDS-PAGE. DLL4 was detected using anti-DLL4 (abcam7280) 1:1000 and membranes were re-probed with anti-β-Actin antibody (Clone AC-15; Abcam) 1:100000. Band intensities of DLL4 and β-Actin Western blots were quantified with the Image Lab™ Software (BioRad). DLL4 intensity was adjusted for levels of β-Actin and blotted relative to band intensity of the control littermate.

Phenotypic analysis of post-natal retinal angiogenesis

Total number of branch points, tip cells and retinal outgrowth length were measured with ImageJ software from pooled images of retinas from at least two independent litters. The ImageJ plugin "AnalyzeSkeleton" by Ignacio Arganda Carreras was use to perform branching analysis of IB4 staining of images acquired with a Zeiss 710 confocal microscope. Tip cells were counted and marked manually with the ImageJ cell counter and the outgrowth length was measured for all quadrants of each retina with the straight line tool.

Generation and analysis of transgenic zebrafish

F0 transient mosaic transgenic zebrafish embryos were generated by the Tol2 system as previously described (Sacilotto et al. 2013). Embryos were maintained in E3 medium (5 mM NaCl, 0.17 mM KCl, 0.33 mM $CaCl_2$, 0.33 mM MgSO4) at 28.5 °C. Enhancer activity was scored by analysing GFP reporter expression on anaesthetised live embryos between 24 and 32 hpf with Zeiss 710MP confocal microscope, kept in a heated chamber at 28.5°C.

Electrophoretic mobility shift assay

Electrophoretic mobility shift assay (EMSAs) were performed as described previously (Sacilotto et al., 2013). Proteins were made using the TNT Quick Coupled Transcription/Translation system as described in the manufacturer's directions. The ETS1 DNA binding domain (ETSDB) was in the pCITE2 plasmid, and transcribed using T7 polymerase. Etv2 was in the pCS2 plasmid, and transcribed using Sp6 polymerase. Mef2A, Mef2C and Mef2D were in pcDNA3 and transcribed using T7.

To label the probe, double stranded oligonucleotides were labelled with 32P-dCTP, using Klenow (Promega) to fill in overhanging 5' ends, and purified on a non-denaturing polyacrylamide-TBE gel. 20 µl binding reactions consisted of 2-5 µl protein or lysate control and 2 µl 10X binding buffer (40mM KCl, 15 mM HEPES pH 7.9, 1 mM EDTA, 0.5 mM DTT, 5% glycerol). 0.5 µg of poly dI-dC was used. For competitor lanes, a 100-fold excess of competitor DNA was added in a volume of 1µl unless otherwise specified. Binding reactions were incubated at room temperature for 20 minutes before the addition of radiolabeled probed, after which they were incubated an additional 20-40 minutes. Gels were electrophoresed on a 6% non-denaturing polyacrylamide gel.

Neo-angiogenesis assays

For the Matrigel assays, 8 to 10 week-old male Dll4in3:*LacZ* and Dll4in3mutMEF:*LacZ* transgenic mice were subcutaneously injected in the flanks with 400 µl of BD Matrigel Basement Membrane Matrix (BD) supplemented with 2 µg/ml Fibroblast Growth Factor (Peprotech). Matrigel plugs were harvested between 14 days after injection. For the tumour studies, 8 to 10-week old male Dll4in3:*LacZ* and Dll4in3mutMEF:*LacZ* transgenic mice were subcutaneously injected in either

flank with 100 µl of a mixture of BD Matrigel Basement Membrane Matrix (BD) containing $1x10^5$ B16-F10 melanoma cells (50 µl of Matrigel + 50 µl of cells re-suspended in serum-free Dulbecco's Modified Eagle's Medium). Tumours were harvested at the HO established humane end-point of 12mm diameter. In both cases, the samples were fixed in 2% PFA + 0.2% glutaraldehyde in PBS on ice for 20 mins, washed in PBS twice for 20 mins at RT and stained in X-gal staining solution as previously described. After X-gal staining, samples were post-fixed in 4% PFA at 4°C, photographed with a stereo microscope (Leica M165C) equipped with a ProGres CF Scan camera (and ProgRes CapturePro software, Jenoptik), dehydrated and embedded in paraffin. 5 µm microtome sections were counterstained with nuclear fast red for histological analysis or analyzed by immunofluorescence using 1:300 rat α-CD31 antibody (Dianova) and 1:300 Alexa Fluor-488 donkey α-rat IgG (Life Technologies) to visualize vascular content.

Prior to neo-angiogenesis studies all male mice were crossed with WT females to confirm genotype through analysis of X-gal expression in embryonic offspring. To further validate transgene expression in each animal, the bladder was harvested from each adult mouse and subjected to X-gal staining along with Matrigel plug or tumor.

For quantification purposes, one half of X-gal stained matrigel plugs and allograft tumors were imbedded in paraffin and sectioned transversally at 5 µm. X-gal positive vessels were counted in matrigel plugs from Dll4in3:LacZ (N=4) and Dll4in3mutMEF:LacZ transgenic mice (N=5) and tumors from Dll4in3:LacZ (N=3) and Dll4in3mutMEF:LacZ (N=3) mice. X-Gal positive vessels were quantified per surface area of Matrigel plug/tumor.

Immunostaining on mouse tissue and HUVECs

Embryonic hindbrains were dissected and processed as described previously (Fantin et al. 2013). In brief, hindbrains were freshly dissected in ice-cold PBS and subsequently fixed in 2% PFA on ice for one hour. Whole embryos were dissected and fixed in 4% PFA on ice for 1 hour. Tumours were fixed in
4% PFA for 1h on ice, washed in PBS, left in 15% and 30% sucrose in PBS until the tissue sunk to the bottom of the container and embedded in OCT for cryosectioning. For retinal immunostainings, eyeballs were enucleated at the indicated time-points and fixed in 4 % PFA for 1 hour at room temperature (MEF2 immunostaining) or for 90 minutes (DLL4 immunostaining). Human renal clear cell carcinoma samples were snap frozen and stored in liquid nitrogen. Frozen sections were prepared and fixed in 4 % PFA on ice for 10 min. Cultured HUVECs were washed in ice-cold PBS and fixed in 4 % PFA on ice for 10 min. Frozen sections and HUVECs were processed for immunostaining using previously described methods (Nikitenko et al. 2006)

After 1 hour incubation in blocking solution containing (10% Normal Donkey Serum and 0.1% (v/v) TritonX-100 in PBS (PBS-T)), samples were incubated overnight at 4°C with the designated primary antibodies (MEF2A (Abcam ab109420), MEF2C (Cell Signalling 5030s), MEF2D (BD 610774), EP300 (Active Motif 61401), HDAC4 (GeneTex GTX110231), Dll4 (R&D systems, AF1389), Isolectin B4 (Vector Laboratories, DL-1207), Erg (Abcam, ab92513)) in 0.1% PBS-T. Samples were washed in PBS-T and subsequently incubated for 3 hours or overnight (DLL4) with suitable species-specific Alexa Fluor® or Biotin-conjugated secondary antibodies (1:300, Thermo Fisher Scientific) in 0.1% PBS-T at room temperature. Visualisation

and imaging were performed using Zeiss 710 confocal microscope system. The Central Oxfordshire Research Ethics Committee (C00.147; C02.216) and Canterbury Ethics Committee (V2-4 02.06.98-01.05.2002) approved the use of human tissues.

Embryoid Body Assay

The YFP- and dsRed wild-type ES cells were a gift from Holger Gerhardt and described in (Jakobsson et al. 2010). To generate ES cells without functional MEF2A and MEF2C proteins, CRISPR/Cas9 mutagenesis was used. Single guide-RNAs (sgRNAs, see below for sequence) suitable for targeting Cas9 to the coding regions of *Mef2A* were designed against exon 4 (GRCm38/mm10, previously referred to as exon 2), and for Mef2C against exon 4 (GRCm38/mm10, previously referred to as exon 2) using the http://crispr.mit.edu/ search algorithm. sgRNAs were selected on the basis of their predicted off-target activities: in both cases at least 3 nucleotide mismatches are required to achieve a match to additional sites within the genome and all of these putative off-target sites map to intergenic regions, indicating a good level of specificity. These exons encode the second part of the MADS box and the entire MEF2 domain for each proteins, and are the same exons deleted in published *Mef2a* and *Mef2c* targeted null mice (Lin et al. 1998; Naya et al. 2002),. Complimentary oligonucleotides containing these guide-RNA target sequences (see below) were annealed and cloned into the BbsI site of pX330 (Addgene #42230) modified by the addition of a puromycin selection cassette. 1×10^{6} dsRed WT mouse embryonic stem cells were electroporated with 5 µg of the cloned pX330-Puro vectors using the Neon transfection system (ThermoFisher Scientific) (3×1400 V, 10 ms) and plated on cell culture dishes pre-coated with 0.1% gelatin. 24 hours following electroporation, the cells were placed in 600 ng/ml puromycin selection for

48 hours to allow an enrichment for successfully transfected cells. Approximately 8 days after electroporation, individual colonies were isolated, expanded and genotyped. The target gene was amplified (primers listed below) and Sanger sequencing was performed to establish the nature of the mutation. Where ambiguous mixed traces were obtained, the PCR product was cloned into pCR2.1-TOPO (ThermoFisher Scientific) and multiple plasmids sequenced to establish the identity of the individual alleles present. Clones were selected on the basis of harbouring large indel mutations causing a nonsense mutation and a predicted premature translational stop on both alleles of the target genes. Western blots confirmed deletion. For the generation of double knock-out ES cell lines, previously identified single mutant lines were subsequently re-transfected as above with a different plasmid.

sgRNA information: (GRCm38 assembly)

Mef2A, sgRNA: GAGTTCGTCCTGCTTTCATG (chr77:67295388-67295407 (-), guide-RNA cloning oligos (5'-3'): F CACCGAGTTCGTCCTGCTTTCATG, R GGAGGTGTGTGTGATGGATGAAGCTA, Genotyping primers (5'-3'): F AAACCATGAAAGCAGGACGAACTC, R CCGCTTAAACATTAGGTTTGTATGG.

Mef2C, sgRNA: GAGTTTGTCCGGCTCTCGTG (chr 13:83592949-83592968 (-), guide-RNA cloning oligos (5'-3'): F CACCGAGTTTGTCCGGCTCTCGTG, R ACATCTCCAGTTTCCCTGTCTACCAC. Genotyping primers (5'-3'): F AAACCACGAGAGCCGGACAAACTC, R GTTGCAGCCATAGATGGGGTAAACG

All ES cell clones were cultured in Knockout DMEM (ThermoFisher Scientific) supplemented with 2 mM L-Glutamine (Sigma), 1×non-essential amino acids (Sigma), 0.1 mM β-mercaptoethanol (Sigma), 1000 U/ml ESGRO (Millipore) and

10% fetal bovine serum (ThermoFisher Scientific) on a layer of irradiated mouse embryonic fibroblasts (DR4) in the presence of leukaemia inhibitory factor (LIF). Embryoid bodies were generated as previously described (Jakobsson et al. 2006). Briefly, prior to *in vitro* differentiation, ES cells were cultured for at least two passages in the above media supplemented with CHIR99021 (3 µM), and PD0325901 (1 µM) in the absence of feeders layers on 0.1% gelatin coated culture dishes. Cells were trypsinized, depleted of LIF, mixed with another strain in case of competition and left in suspension as hanging drops (day 0). On day four the formed embryoid bodies were transferred to a polymerized collagen I gel with addition of 30ng/ml $VEGFA_{164}$ (Peprotech). Medium with or without $VEGFA_{164}$ was changed on day six and every day thereafter.

HUVEC spheroids

HUVECs, previously transfected with control or Mef2A, C and D siRNA and expressing GFP and dsRed were trypsinized and endothelial cell spheroids were generated using established protocol (Korff 1998). Equal number of endothelial cells (750 cells/spheroid) were suspended in 20µl of culture medium containing 0.25% carboxymethyl-cellulose and culturing at 37°C (5% CO_2, 100% humidity) to form a single spheroid per well. Spheroids were embedded into collagen gels and stimulated with 50µg/ml $VEGF_{165}$ (R&D). After 24 hours, the sprouting was recorded digitally using confocal microscopy, with at least 10 spheroids per experimental group and experiment.

HDAC inhibitor assays

HUVECs: HUVECs (pooled donors, Lonza) were cultured in full EGM-2 medium (Lonza) at 37°C, 5% CO_2, seeded in 6 well-plates at 100.000 cells/well and used up to the forth passage. Fresh EGM-2 medium was added 24 hours after seeding and replaced after 12 hours by EBM-2 medium. 400 nM Trichostatin A (TSA), 10 µM BML-210 (Abcam), 10 µM MC-1568 (Sigma) or DMSO were added for the indicated time after serum starvation in EBM-2, and harvested with or without 1h VEGF stimulation (EBM-2 + 25ng/ml VEGF165) for RNA extraction Cells were washed in PBS and RNA was extracted with the Illustra RNAspin Mini Kit (GE Healthcare Life Sciences) following the manufacturer's instructions, and used for retro-transcription (Superscript III, Life Technologies) and qRT-PCR (StepOne Plus, Life Technologies) using pre-designed Taqman assays (Applied Biosystems).

Embryo culture: Pregnant C57BL/6 mice crossed with transgenic males were humanely sacrificed and E9.5 embyos dissected in warm Dubelcco's Modified Eagle Medium (DMEM) supplemented with 1% FBS, penicillin (100 U/ml), streptomycin (100 U/ml) and 2 mM L-glutamine, under sterile conditions. Embryos with visible heartbeats were incubated at 37°C in 5% CO_2 for 17 hours in 24-well tissue-culture plates supplemented with either 100µM Trichostatin A (Sigma) or DMSO. Embryos with visible heartbeats were fixed in 2% PFA, 0.2% glutaraldehyde in PBS on ice for 20 mins, and stained in X-gal staining solution as described.

In utero intracerebral injections: Exposure of living embryos was performed as previously described for *in utero* electroporation (Garcia-Moreno et al. 2014). Briefly, E12.5 pregnant mice were anesthetized and the uterine horns were exposed, the brains were distinguished and the fourth ventricle injected with a glass microcapillary. Each embryo was injected with an approximate 1µl volume of 10 µM

Trichostatin A (TSA) or DMSO control, dyed with fast green. Embryos were returned to the abdominal cavity and harvested 24 hours after injection.

Chromatin immunoprecipitation (ChIP)

ChIP assays were performed as described in (Sacilotto et al. 2013) with the following modifications: sub-confluent HUVECs (pooled, Lonza) up to the forth passage, growing at 37°C and 5% CO_2 in full EGM-2 medium (Lonza) were starved for 18 hours in EBM-2 medium (Lonza) and then stimulated for one hour with EBM-2 supplemented with 25ng/ml $VEGF_{165}$ for 1 h. Cells were cross-linked with 1% formaldehyde at room temperature for 8 minutes, and the crosslinking reaction was quenched with 0.125 M glycine. Cells were collected in ice-cold PBS, lysed in cell lysis buffer (10 mM NaCl, 3 mM $MgCl_2$, 30 mM sucrose, 10 mM EDTA, 0.5% Nonidet P-40, 10 mM Tris-HCl, pH 7, supplemented with protease inhibitor cocktail), and the resulting nuclei were lysed in nuclei lysis buffer (10 mM EDTA, 1% SDS, 50 mM Tris-HCl, pH 8.1). Chromatin was sonicated using the Covaris S220 sonicator (8.5 minutes, 160 W peak incident power, 5% duty cycle and 200 cycles per burst, at 4°C) to obtain populations of fragments with an average size of 250 bp. The size of the resulting chromatin fragments was analyzed after each independent sonication as follows: chromatin formaldehyde crosslinks were reversed at 65°C in the presence of proteinase K (Fisher Scientific) over-night, and the DNA purified using the PCR purification kit (Qiagen) following the manufacturer's instructions. The resulting purified DNA fragments were analysed by agarose gel electrophoresis.

Ten-fold diluted chromatin was pre-cleared with BSA- (Sigma) and tRNA- (Ambion) saturated Dynabeads protein-G(Life Technologies) for 2 hours at 4°C. Pre-cleared

chromatin was then incubated over-night with 3µg of the corresponding antibodies and Isotype IgG controls, in low-binding tubes (Thermo Fisher Scientific), and 1/10 of the diluted chromatin used in the IP was kept as an Input sample, for each condition. Saturated Dynabeads protein-G(Life Technologies) were added to the samples and incubated for 2 hours at 4°C. Immuno-complexes were recovered with a DynaMag-2 magnet (Thermo Fisher Scientific), extensively washed twice with low salt buffer (20mM Tris-HCl (pH 8.0), 150mM NaCl, 2mM EDTA, 1% Triton X-100, 0.1% SDS), twice with high salt buffer (20mM Tris-HCl (pH 8.0), 500mM NaCl, 2mM EDTA, 1% Triton X-100, 0.1% SDS), twice in LiCl buffer (10mM Tris-HCl (pH 8.0), 250mM LiCl, 1mM EDTA, 1% sodium deoxycholate,1% NP-40), once with TE (pH 8.0) and eluted twice with 100µl of elution buffer (EDTA 10mM, SDS 1%, 50mM Tris–HCl) at 65°C for 10 min. The resulting 200µl were incubated at 65°C for 1 hour with RNAse A (Sigma) followed by incubation with proteinase K (Fisher Scientific) at 65°C over-night. DNA was purified by PCR purification kit (Qiagen) following the manufacturer's instructions, and used as a template for qPCR.

Lentivirus production and infection of HUVEC

Vesicular stomatitis virus-G envelope-pseudotyped lentiviral virions were produced by cotransfecting 2 µg lentiviral (pSicoR-Ef1a-mCherry-Puro or pGIPz-GFP-Puro; from Addgene and Open Biosystems respectively) construct, 1.5 µg Gag-Pol (p8.91), and 1.5 µg VSV-G (pMDG) packaging plasmids (Vart et al. 2007)into a 10-cm dish of ~70% confluent 293T cells using the FuGENE (Roche) protocol. Five hours after transfection, the medium was changed, and 48 hours after transfection, the medium containing the lentiviral virions was collected, passed through a 0.45-µm filter, aliquoted and stored at −80°C. New batch of HEK293T cells was transduced with

lentiviral particles to determine the multiplicity of infection (MOI) prior to HUVEC transduction. Lentiviral infections were done by incubating the desired amount of virus preparation with second passage HUVECs for 5 hours, after which the medium was changed. The selection of cells expressing mCherry or GFP was done using 1µg/ml puromycin (Sigma). The expression of mCherry was confirmed by confocal microscopy and flow cytometry using a FACSCalibur flow cytometer (BD Biosciences).

siRNA

Dharmacon siRNAs targeting *MEF2A*, *MEF2C, MEF2D* or the negative control sequence were purchased from GE Healthcare and are listed below. siRNAs were transfected into primary HUVEC expressing GFP (pGIPz-GFP-Puro HUVEC) at a final concentration of 100nM using Oligofectamine reagent (Life Technologies). Stealth siRNAs targeting Mef2a, Mef2c and Mef2d and the Stealth RNAi™ siRNA Negative Control Med GC Duplex #2 were purchased from Life Technologies. siRNA was transfected into bEnd cells at a final concentration of 25nM using Lipofectamine RNAiMAX reagent (Life Technologies). Cells were transfected at 50% confluency in Opti-MEM reduced Serum Medium with GlutamMAX 1 (Life Technologies) and used after 24 hours. siRNA-mediated knockdown efficiency was confirmed by analysing MEF2A/Mef2a, C/c or D/d protein expression by immunoblotting using described above methods. GFP-expressing HUVECs transfected with siRNA targeting MEF2A, C and D or with non-targeting siRNA control were mixed with mCherry-expressing HUVECs to form endothelial spheroids, which were used for the sprouting essays.

HUMAN siRNA

Target	Pool Catalogue Number	Duplex Catalogue Number	GENE ID	Gene Accession	GI Number	Sequence
MEF2A pool	J-009362-05	J-009362-05	4205	NM_005587	5031906	AUACAAAUCACACGCAUAA
	J-009362-06	J-009362-06	4205	NM_005587	5031906	GAUAAUAUGAUGCGGAAUC
	J-009362-07	J-009362-07	4205	NM_005587	5031906	CAUCAAGUCCGAACCGAUU
	J-009362-08	J-009362-08	4205	NM_005587	5031906	GCAAAUAGCUUAGGCAAAG
MEF2C pool	J-009455-05	J-009455-05	4208	NM_002397	19923214	GACAAGGAAUGGGAGGAUA
	J-009455-06	J-009455-06	4208	NM_002397	19923214	UAACACAGGUGGUCUGAUG
	J-009455-07	J-009455-07	4208	NM_002397	19923214	GAAUAACCGUAAACCAGAU
	J-009455-08	J-009455-08	4208	NM_002397	19923214	GAUCAGCAGGCAAAGAUUG
MEF2D pool	J-009884-05	J-009884-05	4209	NM_005920	40254821	GAAAGGGGUUAAUGCAUCA
	J-009884-	J-009884-	4209	NM_0059	402548	AAGAGGAUGC

	06	06		20	21	GGCUUGAUA
	J-009884-07	J-009884-07	4209	NM_005920	40254821	GCAGAGCUCUCCUCCUUAC
	J-009884-08	J-009884-08	4209	NM_005920	40254821	GCAACAGCCUAAACAAGGU
Control pool	D-001810-10	D-001810-01				UGGUUUACAUGUCGACUAA
	D-001810-10	D-001810-02				UGGUUUACAUGUUGUGUGA
	D-001810-10	D-001810-03				UGGUUUACAUGUUUUCUGA
	D-001810-10	D-001810-04				UGGUUUACAUGUUUUCCUA

Mouse siRNA

Mef2a: Stealth siRNAs (Set of 3) MSS206606, MSS206607, MSS206608, catalogue number 132001.

Mef2c: Stealth siRNAs (Set of 3) MSS206609, MSS206610, MSS206611, catalogue number 132001.

Mef2d: Stealth siRNAs (Set of 3) MSS247430, MSS247431, MSS247432, catalogue number 132001.

shRNA

MEF2A, C or D stable knockdown was performed using lentiviral particles containing shRNAmir for *MEF2A, C or D* or non-silencing target, which were produced according to the manufacturer's (Open Biosystems) instructions. Directed against human *MEF2A, C or D* gene or non-silencing shRNAmir pGIPz constructs (all also expressing green fluorescent protein (GFP)) were transfected together with Gag-Pol (p8.91) and VSV-G (pMDG) packaging plasmids into the HEK293T cells to produce lentiviral virions, infect and select GFP-expressing HUVEC, as described above in the lentivirus production protocol. Selection was confirmed by flow cytometry analysis using BD FACSDIVA™ software. To confirm knockdown efficiency, lysates from the stable knockdown cells were assayed for *MEF2A, C or D* mRNA and MEF2A, C or D protein expression by qRT-PCR, immunoblotting and immunofluorescence respectively using described above methods.

Gene Symbol	Sense Sequence	Full Hairpin Sequence
MEF2A	AGGTCACTTTTACAAAGAG	TGCTGTTGACAGTGAGCGCCAGGTCACTTTTACAAAGAGATAGTGAAGCCACAGATGTATCTCTTTGTAAAAGTGACCTGTTGCCTACTGCCTCGGA
MEF2A	AGCTCAACGTTAACAGATT	TGCTGTTGACAGTGAGCGACAGCTCAACGTTAACAGATTCTAGTGAAGCCACAGATGTAGAATCTGTTAACGTTGAGCTGGTGCCTACTGCCTCGGA
MEF2A	CTTATGAATTGATGACTA	TGCTGTTGACAGTGAGCGACCTTATGAATTGATGACTATATAGTGAAGCCACAGATGTATATAGTCA

	T	TCAATTCATAAGGGTGCCTACTGCCTCGGA
MEF2A	CCCACACTAGCTTGCAGAA	TGCTGTTGACAGTGAGCGATCCCACACTAGCTTGCAGAAATAGTGAAGCCACAGATGTATTTCTGCAAGCTAGTGTGGGAGTGCCTACTGCCTCGGA
MEF2C	GGGTTGATGAAGAAGGCTT	TGCTGTTGACAGTGAGCGCTGGGTTGATGAAGAAGGCTTATAGTGAAGCCACAGATGTATAAGCCTTCTTCATCAACCCAATGCCTACTGCCTCGGA
MEF2C	ACAAGAATATGCAAGCAAA	TGCTGTTGACAGTGAGCGAAACAAGAATATGCAAGCAAAATAGTGAAGCCACAGATGTATTTTGCTTGCATATTCTTGTTCTGCCTACTGCCTCGGA
MEF2C	AGGGAAAGTCCCTCAGTCA	TGCTGTTGACAGTGAGCGCAAGGGAAAGTCCCTCAGTCAATAGTGAAGCCACAGATGTATTGACTGAGGGACTTTCCCTTTTGCCTACTGCCTCGGA
MEF2C	CTTGCACTAGCACTCATTT	TGCTGTTGACAGTGAGCGCGCTTGCACTAGCACTCATTTATAGTGAAGCCACAGATGTATAAATGAGTGCTAGTGCAAGCTTGCCTACTGCCTCGGA
MEF2C	CTAACTTGCCCATATTCTA	TGCTGTTGACAGTGAGCGCCCTAACTTGCCCATATTCTAATAGTGAAGCCACAGATGTATTAGAATATGGGCAAGTTAGGATGCCTACTGCCTCGGA
MEF2D	CCCAGTTTTTCTCTAAGAT	TGCTGTTGACAGTGAGCGCCCCCAGTTTTTCTCTAAGATATAGTGAAGCCACAGATGTATATCTTAGAGAAAAACTGGGGATGCCTACTGCCTCGGA
MEF2D	TCTGTTTTTGTGTTCATGT	TGCTGTTGACAGTGAGCGCCTCTGTTTTTGTGTTCATGTATAGTGAAGCCACAGATGTATACATGAACACAAAAACAGAGATGCCTACTGCCTCGGA

MEF2D	CTCTTGCAGTATTCCCATA	TGCTGTTGACAGTGAGCGACCTCTTGCAGTATTCCCATACTAGTGAAGCCACAGATGTAGTATGGGAATACTGCAAGAGGCTGCCTACTGCCTCGGA
MEF2D	TCTATTTTTGTCAAAGTAT	TGCTGTTGACAGTGAGCGCTTCTATTTTTGTCAAAGTATATAGTGAAGCCACAGATGTATATACTTTGACAAAAATAGAATTGCCTACTGCCTCGGA
MEF2D	CTTTGCACGTTGTACACAT	TGCTGTTGACAGTGAGCGAACTTTGCACGTTGTACACATATAGTGAAGCCACAGATGTATATGTGTACAACGTGCAAAGTGTGCCTACTGCCTCGGA

Statistics

Statistical significance was determined by independent two-tailed *t*-test assuming unequal variances.Values are expressed as mean +/- SD or SEM, as indicated.

Imaging acquisition and analysis

Confocal images were acquired with a Zeiss 710 MP confocal microscope. For live imaging of zebrafish embryos samples were kept at 28.5°C. Live embryoid bodies were kept at 37°C and 5% CO_2 during the entire imaging process.

<u>Embryoid body analysis</u>: Area of embryoid bodies was analysed with ImageJ using z-stacked images.

<u>Spetral un-mixing and pseudocolour images:</u> Un-mixing of X-Gal in retina and hindbrain bright field images was done using the spectral un-mixing for ImageJ, plugin by J. Walter. Briefly, sample colour images showing only X-Gal (blue) or background (red/brown) were collected in RGB format and used to define the un-

mixing matrix. The individual colour channels were separated and X-Gal only regions used to define the X-Gal contribution over the red, green and blue channels. A region of background only was used to define its contribution over the three colour channels. Inverted colour channels were separated before applying the algorithm which un-mixes the X-Gal into one image and the background into another, which is discarded. The Unmixed X-Gal image was merged with the corresponding fluorescent channel image, with a maximum intensity projection of the fluorescent z-stack to allow for the greater depth of focus of the bright field image.

Bioinformatic analysis of MEF2-enriched binding sites

MEF2C and corresponding H3K27Ac ChIP Seq data was obtained using publically available ChIP-seq data from (Maejima et al., 2014), data accessible at NCBI GEO database (Edgar, 2002), accession numbers GSE32547, GSE32644, GSE32693 and GSE41553. Raw reads were trimmed with Sickle, and duplicate PCR reads removed with rmdup. Reads were then aligned to human genome build hg19 using Bowtie2, and peaks were called with MACS2. Expression data of tip-cell enriched genes were obtained from (del Toro et al. 2010) and (Strasser et al. 2010). Mouse genes/loci were converted to human orthologs using MGI (http://www.informatics.jax.org/batch), Ensembl BioMart (http://www.ensembl.org/biomart/martview/2595738f714a41407e96a6fb2aade7b4), bioDBnet (http://biodbnet.abcc.ncifcrf.gov/db/db2db.php), and UCSC TransMap (https://cgwb.nci.nih.gov/cgi-bin/hgTrackUi?g=transMapAlnUcscGenes&hgsid=9701168).

For the enrichment analysis, RefSeq genes were obtained from UCSC, and anti-sense genes were removed. MEF2C peaks were required to reside < 500bp from a

H3K27Ac peak and < 200kb from a RefSeq gene. Fisher's Exact was used to determine enrichment of MEF2C peaks in or around genes upregulated in tip cells.

Taqman probes

Dll4in3 Chip Custom Taqman probe (FAM) TCCTGCGGGTTATTTTTGGCGTGGG

Dll4in3 ChIP F3 GTCGGACGCTCGGATTC

Dll4in3 ChIP R3 GAGCACGGCGGTGAGAAAG

Dll4 Taqman expression assay (FAM) Hs00184092_ml

ACTB Taqman expression assay (FAM) Hs01060665_ml

CDH5 Taqman expression assay (FAM) Hs00901465_ml

Mef2A Taqman expression assay (FAM) Hs01050409_ml

Mef2C Taqman expression assay (FAM) Hs00231149_ml

Mef2D Taqman expression assay (FAM) Hs00954735_ml

Genotyping primers

HSP68LacZ1 (genotyping) CAAGCGAAGACAAGAGAAGCAGAGCGAGC

HSP68LacZ2 (genotyping) AAACCAGGCAAAGCGCCATTCGCCATTCAG

Internal012 (genotyping) TGGACAGGACTGGACCTCTGCTTTCCTAGA

Internal013 (genotyping) TAGAGCTTTGCCACATCACAGGTCATTCAG

EMSA primers

MLC Mef2 control F (Esser et al.)

ctagGATCCTCATCTTTTAAAAATAACTTTTCAAAAG

MLC Mef2 control R (Esser et al.)

ctagCTTTTGAAAAGTTATTTTTAAAAGATGAGGATC

Dll4in3 MEF2 WT F (Sacilotto et al. 2013)

ctagCCTGCGGGTTATTTTTGGCGTGGGA

Dll4in3 MEF2 WT R (Sacilotto et al. 2013)

ctagTCCCACGCCAAAAATAACCCGCAGG

Dll4in3 MEF2 MT F:ctagCCTGCGGGTcgcccTTGGCGTGGGA

Dll4in3 MEF2 MT R:ctagTCCCACGCCAAgggcgACCCGCAGG

HLX-3 MEF2-a WT F: ctagGTATATTTATATTTTCATTGCTAG

HLX-3 MEF2-a WT R: ctagCTAGCAATGAAAATATAAATATAC

HLX-3 MEF2-a MT F: ctagGTATATTTACGCCCTCATTGCTAG

HLX-3 MEF2-a MT R: ctagCTAGCAATGAGGGCGTAAATATAC

HLX-3 MEF2-b WT F: ctagTGCAGTATCCTAAAATACTAAGGCCGC

HLX-3 MEF2-b WT R: ctagGCGGCCTTAGTATTTTAGGATACTGCA

HLX-3 MEF2-b MT F: ctagTGCAGTATCCTAGGGCGCTAAGGCCGC

HLX-3 MEF2-b MT R: ctagGCGGCCTTAGCGCCCTAGGATACTGCA

HLX-3 MEF2-c WT F: ctagGGCCGCTTCTATTTTGAGACCAATCT

HLX-3 MEF2-c WT R: ctagAGATTGGTCTCAAAATAGAAGCGGCC

HLX-3 MEF2-c MT F: ctagGGCCGCTTCCGCCCTGAGACCAATCT

HLX-3 MEF2-c MT R: ctagAGATTGGTCTCAGGGCGGAAGCGGCC

hlx-3 MEF2-a WT F: ctagAGTTAATTGTGTTTTCGTTTCAGCGG

hlx-3 MEF2-a WT R: ctagCCGCTGAAACGAAAACACAATTAACT

hlx-3 MEF2-a MT F: ctagTGTTATGTCTATTAGTTGTTCAGCGG

hlx-3 MEF2-a MT R: ctagCCGCTGAACAACTAATAGACATAACA

hlx-3 MEF2-b WT F: ctagCTGTATCCTAAAATACTAAAGCAT

hlx-3 MEF2-b WT R: ctagATGCTTTAGTATTTTAGGATACAG

hlx-3 MEF2-b MT F: ctagCTAATACAATATCTTGCAAAGCAT

hlx-3 MEF2-b MT R: ctagATGCTTTGCAAGATATTGTATTAG

hlx-3 MEF2-c WT F: ctagAAAGCATCTTCTATTTTTGGCCTAAA

hlx-3 MEF2-c WT R: ctagTTTAGGCCAAAAATAGAAGATGCTTT

hlx-3 MEF2-c MT F: ctagAAAGCATTCTTCTTATTTGGCCTAAA

hlx-3 MEF2-c MT R: ctagTTTAGGCCAAATAAGAAGAATGCTTT

Enhancer sequences

<u>Dll4in3 (mouse)</u>

CTGCTGATATCGCTATCTCTAATGTCCCCCACCCCCCCTTTTGCTTCCCAGGGA

ACCTTCTCACTCAACATCCAAGCTTGGCACACACCGGGAGACGACCTGCGGCC

AGGTGAGTATCTAACTTCTCGGCCACAGGGGGGCGACATCACACAGCGCCGAA

AGAGTTAACCAGTTATAGGCGGGGGTGGGGGTTGGGGACGCAGGCTTGGGGG

GTGGGGGCCAGGACGCTTAGCTTGGCCGGAGCTGCGCCCCGCGCTGGACGC

TCGGATTCCGCTCGCTGCCTGGACTCAGAGCACAATTGCGTTTCCTGCGGGTT

ATTTTTGGCGTGGGAACGCGGGGAGCACGGCGGTGAGAAAGGCCGAGGCTGC

CAGCGCCGCTGACGGGCCTCTTCCTGTATTTTACACCTTTTGCGAATTCCGCTC

CTTTGGAAAGGGAATAATGGCTTTGGGATGTTGTTCTGACACAGAGGAAAAGGA

TATTTCACCAGCACAACAATTCTCACTTTGAAAAGGAAAAAGAAAAACCATTACC

TACGTCTAGAACAGAACCCCTTGCTCCCAGTTCTCGAACCAGAAAACTTCCCCC

TTTAAATTTTTTCTTTTTTTCCATTTTGACCTCTTTTCCTCTTTCCCCTCCGTATCT

GCCTCCACAACCCTAGGATATCTTAACATCCGTCCATTGTACCCTTTTTTGAATG

CTATCAAGCCCCCTGCACATGCACACACCCAGGGAGACTAAGTAGCAAGATTC

TGGGACCCTCTGGCCTGTGCTTACTTGCAGGTAGAGTTAATCTAGATAATTAGA

GTGTGAACTGACCACCATAGTCACAACTAAAGAGAGAGTTGGCAGCAGTCAACT

CTCTCTGAATCAGGTTGGCTTTCTGAATCAGGTTCTCTGACCAAAGCCTCTTTCT

GCAGAGACTTCGCCAGGAAACTCT

Dll4in3mutMEF (mouse)

CTGCTGATATCGCTATCTCTAATGTCCCCCACCCCCCCTTTTGCTTCCCAGGGA

ACCTTCTCACTCAACATCCAAGCTTGGCACACACCGGGAGACGACCTGCGGCC

AGGTGAGTATCTAACTTCTCGGCCACAGGGGGGCGACATCACACAGCGCCGAA

AGAGTTAACCAGTTATAGGCGGGGGTGGGGGTTGGGGACGCAGGCTTGGGGG

GTGGGGGCCAGGACGCTTAGCTTGGCCGGAGCTGCGCCCCGCGCTGGACGC

TCGGATTCCGCTCGCTGCCTGGACTCAGAGCACAATTGCGTTTCCTGCGGGTcg

cccTTGGCGTGGGAACGCGGGGAGCACGGCGGTGAGAAAGGCCGAGGCTGCC

AGCGCCGCTGACGGGCCTCTTCCTGTATTTTACACCTTTTGCGAATTCCGCTCC

TTTGGAAAGGGAATAATGGCTTTGGGATGTTGTTCTGACACAGAGGAAAAGGAT

ATTTCACCAGCACAACAATTCTCACTTTGAAAAGGAAAAAGAAAAACCATTACCT

ACGTCTAGAACAGAACCCCTTGCTCCCAGTTCTCGAACCAGAAAACTTCCCCCT

TTAAATTTTTTCTTTTTTTCCATTTTGACCTCTTTTCCTCTTTCCCCTCCGTATCTG

CCTCCACAACCCTAGGATATCTTAACATCCGTCCATTGTACCCTTTTTTGAATGC

TATCAAGCCCCCTGCACATGCACACACCCAGGGAGACTAAGTAGCAAGATTCT

GGGACCCTCTGGCCTGTGCTTACTTGCAGGTAGAGTTAATCTAGATAATTAGAG

TGTGAACTGACCACCATAGTCACAACTAAAGAGAGAGTTGGCAGCAGTCAACTC

TCTCTGAATCAGGTTGGCTTTCTGAATCAGGTTCTCTGACCAAAGCCTCTTTCTG

CAGAGACTTCGCCAGGAAACTCT

Dll4-12 (mouse)

TCCTAAGTCCTCCCTGTTCTGAAATGTCCAGGCTGCAGATCCCCCTGGGT

ACCTAGGTGGCCAGGCCTGCAGCACCCTCCCCTACAGACAGGGTGACGAC

ATTGTTGTTCTTATACTACAGGGCCTTCCGCCTGAGGTTCCGGCTCCTAA

ATGGTGGGAATAAGGGTCCCCAGGACAAAGCCGGCCTGGTTCCCGCAGTC

ACCTCAGAATGGACGGAATCCCCATTGTGTATGGTCGCCCCATGCCCGCC

CTCACTTACCCTCACCGGACATGCCAACAAACAGCTCATTGAGCCTGGGG

AGGGGCCGGGGAGACAACAATGCCCCCCAGAAGGCAATGGTAGTGAGCTC

AGCTGGGGGTGGAGGCTATCATGGAGAGCGATGATCTCAGCCTTGGGTGA

GCTGGCCACTGTGACAGGCTGACCTGAGCCTGGCACCCCAGTGAGGCTGA

GAAAGGCTGACCCTGCAGCCTCACTCTGACTGCCTGGGAACAGACCAGGA

CTTCCCATATCCTTAAGCTGGCCCTGTCCTGAGTGCCAAGCAGGTGCAGC

TGGCAGGTCCTTAAACGCAGGAGAGATATCTTGGACCCAAGAGATTGGTG

GACACTTCCTCTGCTGTTCCATTCCAGAAAACATCATGGAGGGTCAGAAC

AAACAGCTCTCCTATACCAGCTGCCCACACTGATCACAGTAGAACTTATG

GATCAACCAGCAAATGTGCTTTCTATGACCAAAGCGTCTAAAGAGACTGG

GATAGTCTTGCAAAGTGTTGAAACCTAGGCAGGAGGAATTCAAGCCCTTA

AACAGCCCAGAACTGATCTTAGTGTTCCTGAGAGCTCACCTAACATTAAG

ETS1+194 (human)

GAGCATTGATGAAGCAGCTCGATGAACTCTTGTAAGACCTTTCTCCAAGTGAGT

TATCATTCCAAGGTTCCTGTTGTTACTTTCTGAAGGGCTTTGTGCTTATGAAGCA

GATAGGGACAGAAGGGCGGGCCGGACCCCATGGTCATCTGGCAGCCTTGCTT

GTGTTGGGTCAGCGTGCCAGCATTCATGCCCCTGGGGGTGTGAGCACCCTTTC

TGGAATCTTGAAAGGAAATGCAACCTCCCCAAACCCCACTACTCTCTCAAGGAG

TCATGGGTGATGAAGATGTTTCCTGCCATTCTCTGCAGGACAGAGAGTTGCTTA

ACCCTCGTTTCATTATCAGTGGAGGAGCCAGCCATTGGACCCCCACCCCTCAG

AGTGAGCCAGCAGCCAGAACTAGCCCTTTGGCATCCCCCGTCTTGATTGCAAG
CAAGCAAACCTGTCACCTTCCTGTTTTATCTACTCATGGTGTTTGTTTCCTTTGA
CGTGGATTCTTACACTTAGGGTGGGAGGGATTTCTCGAAAAACATTTGTTGTAT
GCCACTGATCCTGGCCCCACAACGCCCCTTTCTCTTTCCTTTTCAAGGCTCCAG
GAATTGCTAAATGGCATATCCTGAACACCCAGCACTCTGGGGCTAAAAATACCA
GAGGGTGTGTTTATCTAGATTGGGGCTTCCTCTCCAGGGGCTTTACAGACGTTA
TCTCATTTATCCTTGAAAACACTCTGCGAGGCAGCATGTAGTCAAATAACTTTAC
CCCAGAAAGGTACGGAAGGCTTGAAGAGGAAAGGTCATCGGTGGAGGTTCTAT
TCTGAGGCCCAGTAGACAGGCTCAGGTCTTTAATTTCTCAGCTT

ELK3-29

GAGCATTGATGAAGCAGCTCGATGAACTCTTGTAAGACCTTTCTCCAAGTGAGT
TATCATTCCAAGGTTCCTGTTGTTACTTTCTGAAGGGCTTTGTGCTTATGAAGCA
GATAGGGACAGAAGGGCGGGCCGGACCCCATGGTCATCTGGCAGCCTTGCTT
GTGTTGGGTCAGCGTGCCAGCATTCATGCCCCTGGGGGTGTGAGCACCCTTTC
TGGAATCTTGAAAGGAAATGCAACCTCCCCAAACCCCACTACTCTCTCAAGGAG
TCATGGGTGATGAAGATGTTTCCTGCCATTCTCTGCAGGACAGAGAGTTGCTTA
ACCCTCGTTTCATTATCAGTGGAGGAGCCAGCCATTGGACCCCCACCCCTCAG
AGTGAGCCAGCAGCCAGAACTAGCCCTTTGGCATCCCCCGTCTTGATTGCAAG
CAAGCAAACCTGTCACCTTCCTGTTTcgTCTACTCATGGTGTTTGTTTCCTTTGAC
GTGGATTCTTACACTTAGGGTGGGAGGGATTTCTCcgcccACATTTGTTGTATGC
CACTGATCCTGGCCCCACAACGCCCCTTTCTCTTTCCTTTTCAAGGCTCCAGGA
ATTGCTAAATGGCATATCCTGAACACCCAGCACTCTGGGGCcgcccATACCAGAG
GGTGTGTTTATCTAGATTGGGGCTTCCTCTCCAGGGGCTTTACAGACGTTATCT
CAcgTATCCTTGAcgACACTCTGCGAGGCAGCATGTAGTCAAATAACTTTACCCC

AGAAAGGTACGGAAGGCTTGAAGAGGAAAGGTCATCGGTGGAGGTTCTATTCT

GAGGCCCAGTAGACAGGCTCAGGTCTTTAATTTCTCAGCTT

HLX-3WT human (short sequence for HLX-3:GFP transgene)

CAGAACCAAGAAAGCAGCAGGAAACCCTGGGCTCGCCGGAGTTCAGGTCATCT

CCTGTTTTTTGATGGGCTCAAACTCGGGACTAAATGAGCGGATGTGCCTGCGA

GATTGGTCTCAAAATAGAAGCGGCCTTAGTATTTTAGGATACTGCAGAAATCCTT

ATTTCTTCCGGAGAAAAACTCTATAATGACTCCTAGCACTTCCCCTTTCAATATT

TGTGCAACTTTATCTCCACCGGCAAGTTGCTCGGGTGACGCAAATCCGCCCTTA

TGTATTACAACTACCAAAATAAATGCACGGAAATCAATTCCCTTCTAGCAATGAA

AATATAAATATACAGCTGCGAC

HLX-3WT human (long sequence HLX-3:*LacZ* transgene)

CTGCAAACATTTAGACCTCAGCCTAAGCCCAAACTCACGGTTGTGAAAGAGGTG

CATTTACTCCTTACCCGAGGCAGCGCCGCGCGCCCTCACCCTCTCTCTTAAGA

AATAAGACTCCCCTATCAGCTTTCACCCTTTCCAAGGTTTGCGACGCTCCGGGG

TGGGCACGGGATACAGACCCGGGGGAATGCGAGTCCTCAGGGTTCCCTAGGT

GCCCACAACTCGCATCTGCCGAGATTCAAGGCCCGGAGCGCTGGGCAGATGC

GGCGAAAGCGAGGCCCCGAGGCCCGCCGACTCCTCGCGGCGCCGCGAGCCT

CCCTTTCTCCGCCAGGCCCGCGGCTGGAGTGCAAGTTGCAACTTCGCCTGCGC

GCCCCTTGCTCTCCCAGGCCTCAGCTTCCTCGCAACCGCGTCGGCGCCATCCA

AGGCCTTACTCCCAGCTCCCGGAGGCAAACGCGAAATACAAGTCTTTGGAGCG

ACCCCCGCCGCCCCCTCTACCCCTTCCCCAGGCCCAAGCCTGGCCCCTGCAG

GCCCCTCGCCGAGTTCTGAGCCCGCAGACTCCCGCCCTCCTTACTCCCCACTC

TTCAGAACCAAGAAAGCAGCAGGAAACCCTGGGCTCGCCGGAGTTCAGGTCAT

CTCCTGTTTTTTGATGGGCTCAAACTCGGGACTAAATGAGCGGATGTGCCTGCG
AGATTGGTCTCAAAATAGAAGCGGCCTTAGTATTTTAGGATACTGCAGAAATCC
TTATTTCTTCCGGAGAAAAACTCTATAATGACTCCTAGCACTTCCCCTTTCAATA
TTTGTGCAACTTTATCTCCACCGGCAAGTTGCTCGGGTGACGCAAATCCGCCCT
TATGTATTACAACTACCAAAATAAATGCACGGAAATCAATTCCCTTCTAGCAATG
AAAATATAAATATACAGCTGCGACTTTCCCCTTTAATCTTTGCTTTCCGAGAGGC
TCCGGGCTGTGGCGAAATGAACTGGCCCAGAGCTACTGAGCCGGGCAGGGAG
GTCCTGGGGGTAGAGGCGAGGGCTCCCCAGGGGCTGGTAGCCATGGGGGTT
GAGAATTTAACAGGAAAGAAAAGGGCCTGGCTAAACCGAATTAACTTGAGCTTG
GTCTACGCAGCGGAGA

HLX-3mutMEF

CTGCAAACATTTAGACCTCAGCCTAAGCCCAAACTCACGGTTGTGAAAGAGGTG
CATTTACTCCTTACCCGAGGCAGCGCCGCGCGCCCTCACCCTCTCTCTTAAGA
AATAAGACTCCCCTATCAGCTTTCACCCTTTCCAAGGTTTGCGACGCTCCGGGG
TGGGCACGGGATACAGACCCGGGGGAATGCGAGTCCTCAGGGTTCCCTAGGT
GCCCACAACTCGCATCTGCCGAGATTCAAGGCCCGGAGCGCTGGGCAGATGC
GGCGAAAGCGAGGCCCCGAGGCCCGCCGACTCCTCGCGGCGCCGCGAGCCT
CCCTTTCTCCGCCAGGCCCGCGGCTGGAGTGCAAGTTGCAACTTCGCCTGCGC
GCCCCTTGCTCTCCCAGGCCTCAGCTTCCTCGCAACCGCGTCGGCGCCATCCA
AGGCCTTACTCCCAGCTCCCGGAGGCAAACGCGAAATACAAGTCTTTGGAGCG
ACCCCCGCCGCCCCCTCTACCCCTTCCCCAGGCCCAAGCCTGGCCCCTGCAG
GCCCCTCGCCGAGTTCTGAGCCCGCAGACTCCCGCCCTCCTTACTCCCCACTC
TTCAGAACCAAGAAAGCAGCAGGAAACCCTGGGCTCGCCGGAGTTCAGGTCAT
CTCCTGTCCCCCGATGGGCTCAAACTCGGGACTAAATGAGCGGATGTGCCTGC

GAGATTGGTCTCAGGGCGGAAGCGGCCTTAGCGCCCTAGGATACTGCAGAAAT
CCTTATTTCTTCCGGAGGGGGGCTCTATAATGACTCCTAGCACTTCCCCTTTCA
ATATTTGTGCAACTTTATCTCCACCGGCAAGTTGCTCGGGTGACGCAAATCCGC
CCTTATGTATTACAACTACCAGGGCGAATGCACGGAAATCAATTCCCTTCTAGC
AATGAGGGCGTAAATATACAGCTGCGACTTTCCCCTTTAATCTTTGCTTTCCGA
GAGGCTCCGGGCTGTGGCGAAATGAACTGGCCCAGAGCTACTGAGCCGGGCA
GGGAGGTCCTGGGGGTAGAGGCGAGGGCTCCCCAGGGGCTGGTAGCCATGG
GGGTTGAGAATTTAACAGGAAAGAAAAGGGCCTGGCTAAACCGAATTAACTTGA
GCTTGGTCTACGCAGCGGAGA

hlx-3WT (zebrafish)

CAGCACGCTCTATTTTTGGCACAGCAATGTAAAGTTGTCATGTGCATATGGATG
AAGTATCGCCTCAGTAGTTAATTGTGTTTTCGTTTCAGCGGAGAATAGATTTCCT
CTGTTTATTATAAGCATAAGGACGGATTTGCGTCACCGTGCGACTTGCGAGTTG
AGATAAAGTTGCACAAATATTGAAAAAAGGAAGTGCTAACAGTCATTATAAAGTT
CCACCTCTGTGAGTCGGAGGAAATAAGGGTGCTCTGCTGTATCCTAAAATACTA
AAGCATCTTCTATTTTTGGCCTAAATCTTGCAGGAATATCCGCTCATTTAGCCTG
AATCAGAGCTCATCAAAAACAGGATGACCTGCATCGAGAAACTCCTGTTTCCTA
ATTCCACAGAGCAGAAAACACACACACATACACACACACACGAGTCGGAGTGTT
AGCATTTGAT

hlx-3mutMEF (zebrafish)

CAGCACGCTCTATTTTTGGCACAGCAATGTAAAGTTGTCATGTGCATATGGATG
AAGTATCGCCTCAGTTGTTATGTCTATTAGTTGTTCAGCGGAGAATAGATTTCCT
CTGTTTATTATAAGCATAAGGACGGATTTGCGTCACCGTGCGACTTGCGAGTTG

AGATAAAGTTGCACAAATATTGAAAAAAGGAAGTGCTAACAGTCATTATAAAGTT

CCACCTCTGTGAGTCGGAGGAAATAAGGGTGCTCTGCTAATACAATATCTTGCA

AAGCATTCTTCTTATTTGGCCTAAATCTTGCAGGAATATCCGCTCATTTAGCCTG

AATCAGAGCTCATCAAAAACAGGATGACCTGCATCGAGAAACTCCTGTTTCCTA

ATTCCACAGAGCAGAAAACACACACACATACACACACACACGAGTCGGAGTGTT

AGCATTTGAT

Supplemental References De Val S, Anderson JP, Heidt AB, Khiem D, Xu S-M, Black BL. 2004. Mef2c is activated directly by Ets transcription factors through an evolutionarily conserved endothelial cell-specific enhancer. *Dev Biol* **275**: 424–434.

del Toro R, Prahst C, Mathivet T, Siegfried G, Kaminker JS, Larrivee B, Bréant C, Duarte A, Takakura N, Fukamizu A, et al. 2010. Identification and functional analysis of endothelial tip cell-enriched genes. *Blood* **116**: 4025–4033.

Esser K, Nelson T, Lupa-Kimball V, Blough E. The CACC Box and Myocyte Enhancer Factor-2 Sites within the Myosin Light Chain 2 Slow Promoter Cooperate in Regulating Nerve-specific Transcription in Skeletal Muscle. *jbcorg*.

Fantin A, Vieira JM, Plein A, Maden CH, Ruhrberg C. 2013. The embryonic mouse hindbrain as a qualitative and quantitative model for studying the molecular and cellular mechanisms of angiogenesis. *Nat Protoc* **8**: 418–429.

Garcia-Moreno F, Vasistha NA, Begbie J, Molnar Z. 2014. CLoNe is a new method to target single progenitors and study their progeny in mouse and chick. *Development* **141**: 1589.

Jakobsson L, Franco CA, Bentley K, Collins RT, Ponsioen B, Aspalter IM, Rosewell I, Busse M, Thurston G, Medvinsky A, et al. 2010. Endothelial cells dynamically compete for the tip cell position during angiogenic sprouting. *Nat Cell Biol* **12**: 943–953.

Jakobsson L, Kreuger J, Holmborn K, Lundin L, Eriksson I, Kjellén L, Claesson-Welsh L. 2006. Heparan Sulfate in trans Potentiates VEGFR-Mediated Angiogenesis. *Dev Cell* **10**: 625–634.

Korff T. 1998. Integration of Endothelial Cells in Multicellular Spheroids Prevents Apoptosis and Induces Differentiation. *J Cell Biol* **143**: 1341–1352.

Lin Q, Lu J, Yanagisawa H, Webb R, Lyons GE, Richardson JA, olson EN. 1998. Requirement of the MADS-box transcription factor MEF2C for vascular development. *Development* **125**: 4565–4574.

Naya FJ, Black BL, Wu H, Bassel-Duby R, Richardson JA, Hill JA, Olson EN. 2002. Mitochondrial deficiency and cardiac sudden death in mice lacking the MEF2A transcription factor. *Nat Med* **8**: 1303–1309.

Nikitenko LL, Blucher N, Fox SB, Bicknell R, Smith DM, Rees MCP. 2006. Adrenomedullin and CGRP interact with endogenous calcitonin-receptor-like receptor in endothelial cells and induce its desensitisation by different mechanisms. *J Cell Sci* **119**: 910–922.

Pitulescu ME, Schmidt I, Benedito R, Adams RH. 2010. Inducible gene targeting in the neonatal vasculature and analysis of retinal angiogenesis in mice. *Nat Protoc* **5**: 1518–1534.

Sacilotto N, Monteiro R, Fritzsche M, Becker PW, Sanchez-del-Campo L, Liu K, Pinheiro P, Ratnayaka I, Davies B, Goding CR, et al. 2013. Analysis of Dll4

regulation reveals a combinatorial role for Sox and Notch in arterial development. *Proc Natl Acad Sci USA* **110**: 11893–11898.

Strasser G, Kaminker J, Tessier-Lavigne M. 2010. Microarray analysis of retinal endothelial tip cells identifies CXCR4 as a mediator of tip cell morphology and branching. *Blood* **115**: 5102.

Vart RJ, Nikitenko LL, Lagos D, Trotter MWB, Cannon M, Bourboulia D, Gratrix F, Takeuchi Y, Boshoff C. 2007. Kaposi's sarcoma-associated herpesvirus-encoded interleukin-6 and G-protein-coupled receptor regulate angiopoietin-2 expression in lymphatic endothelial cells. *Cancer Res* **67**: 4042–4051.

Wang Y, Nakayama M, Pitulescu ME, Schmidt TS, Bochenek ML, Sakakibara A, Adams S, Davy A, Deutsch U, Lüthi U, et al. 2010. Ephrin-B2 controls VEGF-induced angiogenesis and lymphangiogenesis. *Nature* **465**: 483–486.
