# Supplementary material for: MEF2 transcription factors are key regulators of sprouting angiogenesis
Source: Genes Dev. 2016 Oct 15;30(20):2297–309. doi: 10.1101/gad.290619.116 (PMC5110996; doi:10.1101/gad.290619.116)
Supplement: Supplemental Material [file supp_30.20.2297_Supplemental_Tables.pdf]

Supplemental Table 1

Locations of statistically significant MEF2 binding peaks 200bp around tip-cell enriched genes (human genes orthologous to original mouse genes identified by del Toro et al., 2010)

Human Gene Name (HUGO symbol)	Location of MEF2C binding peak (hg19)
ABCB1	
ABCC9	
ABCG2	
ACKR2	
ACKR3	
ACVRL1	
ADAMTS1	
ADAMTS16	
ADAMTS9	
ADGRL4	
ADH1C	
ADM	
AKAP12	
AKAP2	
ANGPT2	
ANTXR2	
ANXA1	
ANXA2	
ANXA3	
ANXA6	
AOC3	
APBB1IP	
APLN	chrX:128,788,328-128,788,527
AQP1	chr7:30,960,780-30,960,979
ARAP3	
ARHGAP18	
ARHGAP25	
ARHGAP29	
ARHGDIB	
ART3	
ASPN	
B2M	
B3GNT2	
BAG3	
BCL6B	
BST2	
C10ORF10	chr10:45,474,368-45,474,567
C1ORF54	
C1QC	
C1QTNF6	
C4ORF32	
C8ORF4	
C9ORF3	
CAPG	
CASP4	
CASP8	
CAST	
CAV1	
CAV2	
CCL2	
CCL3	
CCL4	
CCL7	
CCRL2	
CD109	
CD248	
CD302	
CD38	
CD40	
CDH13	
CEBPB	
CHST1	
CHST11	
CHST14	
CHST7	
CLEC1A	
CLEC2D	
CMTM3	
CNN2	
COBLL1	
COL18A1	
COL1A1	
COL6A3	
COQ10B	
CPD	
CREB3L2	
CREM	
CRIP1	
CSF1R	
CSF3R	
CT55	
CTSS	
CXCL1	
CXCL10	
CXCL2	
CYTH4	
DNASE1L1	
DYSF	
ECM2	
ECSCR	
EFNA1	
EGLN3	
EGR3	
EIF2AK2	
ELK3	chr12:96,558,928-96,559,127

EMCN	
EMID1	
EMILIN1	
EMP1	
EMP3	
ENPP3	
ENTPD1	
EPHB4	
ERG	
ESM1	
ETS1	chr11:128,199,311-128,199,510
ETS2	
F2	
F2R	
F3	
FAM101B	
FAM102B	
FAM111A	
FAM167B	
FAM198B	
FAM212A	
FBP1	
FBXO39	
FCER1G	
FCGR1A	
FGD5	
FHL2	
FHOD1	
FKBP9	
FLI1	
flt1	chr13:28,975,622-28,975,821
FOS	
FOSL2	
FOXC1	
FOXF2	
FUT4	
GBP2	
GBP4	
GBP7	
GCH1	
GCHFR	
GIMAP1	
GIMAP4	
GIMAP5	
GIMAP6	
GJA1	chr6:121,733,082-121,733,281
GMFG	
GNB4	
GNG11	chr7:93,575,036-93,575,235
GNG12	chr1:68,190,690-68,190,889
GPIHBP1	
GPR182	
GPR183	
GRK5	
HCLS1	
HECW2	chr2:197,229,893-197,230,092
HELZ2	
HHEX	
HIGD1B	
HLA-G	
HLX	chr1:221,050,031-221,050,230
HPGD	
HSPB8	
HSPG2	chr1:22,236,576-22,236,775
HVCN1	
IFI27	
IGFBP3	
IL10RB	
IL13RA1	
IL1B	chr2:113,569,670-113,569,869
IL1RL2	
INPP5D	
IPO11	
IQGAP1	
IRF8	
IRX3	
ITGA5	
ITGA6	
ITGAM	
ITGB1	
ITIH5	
ITPR1	
JADE2	
KANK4	
KCNE3	
KCNK6	
KCNQ1	
KDELR3	
KIAA0040	chr1:175,170,254-175,170,453
KLF2	
KLHL4	
KLHL6	
KLK8	
LAMA5	
LAMB1	
LCP1	

LCP2	
LDB2	
LDLRAP1	
LEPR	
LGALS3BP	
LITAF	
LOXL2	
LPP	chr3:187,979,822-187,980,021
LXN	
LY86	
LY96	
LYN	
LYZ	
MAN2A1	
MAP2K3	
MAP4K5	
MARVELD2	
MB21D1	
MDFI	
MDFIC	
MECOM	
MEF2C	
MEOX1	
MLKL	
MMRN1	
MMRN2	
MOGAT2	
MSN	
MTMR11	
MYCT1	
MYH9	
MYO18A	
MYO1C	
MYO1D	
N4BP3	
NEXN	
NFATC1	chr18:77,327,501-77,327,700
NFKBIZ	
NID1	chr1:236,260,479-236,260,678
NID2	chr14:52,488,616-52,488,815
NLRC3	
NOSTRIN	
NOX4	
NTRK2	
OPLAH	
OTOR	
P2RY6	
PALMD	
PARP14	
PDGFB	
PDGFRB	
PF4	
PHLDB2	
PIEZO2	
PLAC1	
PLAUR	
PLK2	chr5:57,755,974-57,756,173
PLP2	
PLVAP	
PPFIBP2	
PRCP	
PREX1	
PRG1	
PRICKLE3	
PRKCH	
PRND	
PROCR	
PRRG1	
PRRG3	
PTGS1	
PTPN18	
PXDC1	
RAC2	
RAD51AP1	
RAPH1	
RASGRP3	chr2:33,661,286-33,661,485
RBMS1	
RCSD1	
RELL1	
RGL1	
RGS1	
RGS4	
RGS5	
RHBDL2	
RHOJ	
RIPK1	
RIPK3	
RNF125	
RPS6KA1	
RPS6KA3	
RTP4	
S100B	
SDCBP2	
SDPR	
SEC24D	
SERPINB6	chr6:2,942,359-2,942,558

SERPINE1	
SGK3	
SH3TC1	chr4:8,193,164-8,193,363
SHE	
SLC16A4	
SLC16A6	
SLC19A3	
SLC35D2	
SLC39A8	
SLC43A1	
SLC43A3	
SLCO2A1	
SMAGP	
SMPDL3B	
SNAI2	
SNAP23	
SNX9	
SOX17	chr8:55,249,984-55,250,183
SOX7	
SPARCL1	
SPATA13	
SPATA6	
SPRY1	
SPTY2D1	
ST3GAL6	
ST8SIA4	
STARD13	
STC1	
SYCP3	
TCAF2	
TCF15	
TDRP	
TES	
TF	
TFPI	
TGFB1	
TGFBI	
TGFBR2	
THBD	
THSD1	
TIMP3	
TLR2	
TLR3	
TM6SF1	
TMEM88	
TNFAIP8L1	
TNFRSF11B	
TNFRSF1A	
TNFSF10	
TOR4A	
TP53I11	
TRIM16	
TRIM27	
TRPC6	
TSPAN18	chr11:44,839,646-44,839,845
TSPAN2	
TUBB6	
TYROBP	
UNC45B	
UNC5B	
UPP1	
VANGL1	
VIM	
VIT	
WDFY4	
WIPF1	
WLS	
ZDHHC20	chr13:21,951,375-21,951,574
ZFP36	
ZFP69	

Supplemental Table 2

Locations of statistically significant MEF2 binding peaks 200bp around tip-cell enriched genes (human genes orthologous to original mouse genes identified by Strasser et al., 2010).

Human Gene Name (HUGO symbol)	Location of MEF2C binding peak
ESM1	
GCN1L1	
KAT6A	
TESC	
SDK1	
ADM	
CHST1	
SLC1A3	
SLC16A6	
GCA	
GRIN2B	
SYT16	
POGZ	
ANGPT2	
SEMA3A	chr7:83,753,706-83,753,905
SNTG2	
PRKCZ	
PLCB1	
CXCR4	
PDHA1	
HOMER1	
IGF1	
KCNE3	
PPP2R5E	
SYNC	
SLC41A1	
VLDLR	
CDKN2B	chr9:22,008,630-22,008,829
MAP2	chr2:210,390,640-210,390,839
IGBP1	
IER3	
TRIP11	
AWAT2	
LPIN3	
VEGFA	
KCNK5	
SSH1	chr12:109,233,449-109,233,648
DAPK2	chr15:64,187,840-64,188,039
TRAPPC2	
RNF121	
GPR83	
IL1B	chr2:113,569,670-113,569,869
ST8SIA3	
NDUFS4	
NUP160	
HSD17B2	
U2AF2	
LCP2	
FAM129A	
YTHDC2	
ZNRF2	
ITGAV	
COL2A1	
FNBP1L	
TCTA	
RSRC1	
BLCAP	
CCND2	
DCC	
KLF5	
MTOR	
PCSK6	
ITGB3	
CH25H	
RAB13	
DEDD	
PTPRO	
MFAP3L	
BMP7	
ARMC8	
PPP1R2	
CDR2	
GCNT1	
GLS2	
SAMD8	
ARHGAP18	
PPP3CC	
CFLAR	

INTU	
RGMB	
ANXA6	
FBXO22	
FCHO1	
PRPSAP1	
IGFBP3	
CROT	
ME1	
TDG	
IGFBP4	
RHBDD2	
LYPLA1	
ATP1B1	
ATP6V1C2	
CUL2	
PDE4B	
HOXB5	
UBE4B	
DISP1	chr1:223,168,023-223,168,222
KRT28	
SEMA3F	
HMGXB4	
RFXAP	
RABGAP1L	
NANOS1	
HCK	
FGF11	
IRF8	
MAPKBP1	
TK2	
ABR	
NUDT21	
LRRC16A	
IFT80	
P4HA1	
CCL25	
UGCG	
PKM	
S1PR2	
COPG1	
TUBA1A	
ACKR3	
CFHR1	
UBXN2A	
FPR2	
TP53I11	
ZNF226	
